# Development of a photocatalytic membrane screening reactor (PMSR) for standardized evaluation of immobilized photocatalytic support materials

**DOI:** 10.1016/j.mex.2026.103938

**Published:** 2026-05-02

**Authors:** Michael S. Leupold, Anam Asghar, Klaus Kerpen, Lukas Fischer, Torsten C. Schmidt

**Affiliations:** a Instrumental Analytical Chemistry, University of Duisburg-Essen, Universitätsstr. 5, Essen 45141, Germany; b Centre for Water and Environmental Research (ZWU), University of Duisburg-Essen, Universitätsstr. 2, Essen 45141, Germany; c Technical Chemistry II, University of Duisburg-Essen, Universitätsstr. 5, Essen 45141, Germany; d IWW Water Center, Moritzstr. 26, Mülheim an der Ruhr 45476, Germany

**Keywords:** Amoxicillin, Chemical actinometry, Heterogenous photocatalysis, Immobilization, Reactor design, UV-Filter

## Abstract

Reliable screening of photocatalytic materials for water purification requires laboratory-scale reactors with well-defined irradiation conditions and reproducible operation. Therefore, in this work, a compact, batch-mode photocatalytic membrane screening reactor (PMSR) designed for rapid, high-throughput evaluation of photocatalytic performance under controlled conditions is presented. The PMSR addresses limitations of larger, circulating-flow systems by enabling flexible configuration, efficient mixing, and precise spectral control of irradiation using long-pass filters to selectively excite photocatalysts while minimizing direct photolysis. Reactor performance was characterized using ferrioxalate actinometry, confirming uniform light distribution and accurate determination of photon flux. As a proof of concept, the photocatalytic degradation of amoxicillin was investigated using TiO₂ powder suspensions and TiO₂-decorated polyethersulfone membranes, revealing a strong dependence of degradation kinetics on membrane surface area. The results demonstrate that the PMSR provides a reliable and versatile platform for rapid screening of photocatalytic materials and operating conditions, supporting accelerated material development and process optimization in photocatalytic water treatment research.

## Graphical abstract

**Figure:**
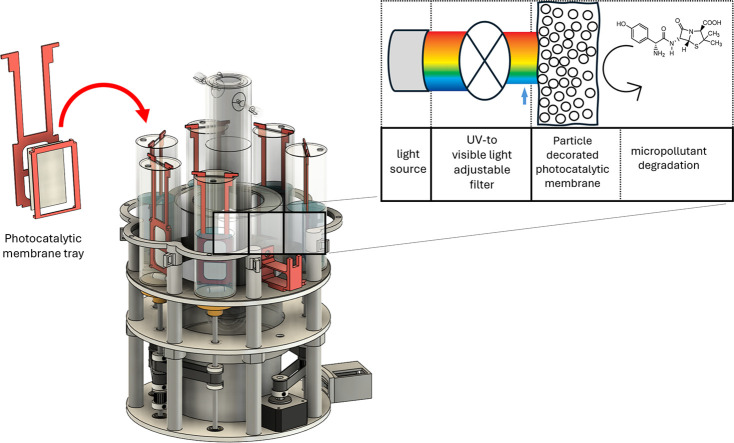

para

**Key feature**

Compact batch-mode high-throughput screening reactor.

Modular design with precise and selective light irradiation control.

High reproducibility ensured by uniform light distribution and efficient mixing.

Alt-text: Unlabelled box dummy alt text

## Specifications table

**Table:** 

**Subject area**	Environmental Science
**More specific subject area**	*Heterogenous photocatalysis on micropollutants*
**Name of your method**	Lab-Scale Implementation of UV long-pass Filters in a Photocatalytic Membrane Screening Reactor (PMSR) to Suppress Direct Photolysis
**Name and reference of original method**	*N/A*
**Resource availability**	*Data will be made available on request.*

## Background

The design of photoreactors plays a critical role in determining the efficiency and reliability of photo(catalytic) water treatment processes. While recent research efforts have predominantly focused on catalyst modification and the optimization and development of large-scale photoreactor systems [1], comparatively limited attention has been devoted to the design of standardized small-scale photoreactors. Such reactors are essential for rapid, reproducible, and cost-effective laboratory screening, as well as for systematic catalyst evaluation and early-stage process development.

Conventional laboratory-scale screening systems, such as merry-go-round photoreactors, are widely used due to their high throughput and operational simplicity, allowing multiple photodegradation experiments to be conducted in parallel [2]. However, these configurations offer limited control over key experimental parameters governing both photolytic and photocatalytic processes. In particular, irradiation conditions are often poorly characterizable, and there is limited spectral selectivity under broad-spectrum irradiation [3]. As a result, direct photolysis of target compounds may occur concurrently with photocatalytic reactions, complicating data interpretation. In addition, the cylindrical geometry of the reaction tubes typically limits effective mixing, resulting in non-uniform photon exposure, inefficient catalyst suspension, and challenges associated with catalyst recovery and reuse. These limitations underscore the need for improved photoreactor designs that provide enhanced control over irradiation conditions while facilitating efficient photocatalyst recovery and reuse.

A recently reported photo(catalytic) reactor design has addressed several of these challenges at a larger scale [4]. A Modular Annular Photocatalytic Membrane Reactor (MAPMR) was developed to achieve uniform light distribution, enable recovery and reuse of immobilized photocatalysts, and provide precise control over key reaction parameters during the photo(catalytic) degradation of micropollutants [4]. The reactor configuration included a chemical filter solution integrated into the cooling path of the irradiation source, enabling selective spectral control. However, the MAPMR was designed as semi-pilot scale system for the evaluation of established immobilized photocatalytic materials under continuous-flow conditions with integrated process monitoring. Therefore, its applicability to rapid screening of photo(catalytic) processes is limited, as such studies require parallel and systematic variation of multiple experimental parameters.

To address these limitations, the present work introduces a ‘photocatalytic membrane screening reactor (PMSR)’**,** specifically designed for systematic and reproducible laboratory-scale screening of photo(catalytic) processes, while drawing on the concepts established in the MAPMR. The novelty of the PMSR lies in its integrated modular design, which enables enhanced flexibility and improved control of irradiation conditions, catalyst configuration, and reactor operation within a unified platform. The PMSR differs from conventional merry-go-around photoreactors by incorporating: (i) a central chemical filter reservoir for selective wavelength control, (ii) support frames for immobilized photocatalytic material; (iii) independent adjustable stirring for uniform mixing; and (iv) integrated spectral monitoring capability. Compared with the MAPMR, PMSR further incorporates: (i) a separate, removable filter reservoir to facilitate rapid exchange of chemical filter solutions; (ii) parallel batch-mode reaction vessels for high throughput process screening; and (iii) removable membrane holders with adjustable positioning inside the reaction vessels. The applicability and performance of the PMSR were evaluated using ferrioxalate actinometry and photocatalytic degradation of amoxicillin, employing both TiO_2_ suspension, and TiO_2_-decorated membranes. Further methodological details are provided below.

## Method details

### Phase 1: reactor description

The PMSR was developed as a laboratory-scale adaptation of the previously reported MAPMR [4]. The reactor incorporates a few key design features of the earlier system in a compact configuration suitable for controlled laboratory operation and systematic screening studies.

**Fig. 1:**
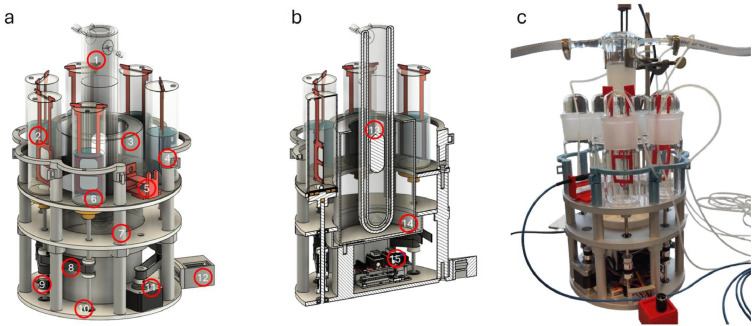
| a) 3D model of the photocatalytic screening reactor (left) and cross-section (right): 1) cooling tube; 2) irradiation vessel with photocatalytic membrane; 3) central UV filter reservoir; 4) irradiation vessel cage; 5) spectrometer optics mount; 6) stir bar; 7) mounting column; 8) timing belt (GT2, 6 mm wide); 9) pulley (GT2, 5 mm inner diameter, 21 teeth); 10) ball bearing (6 mm inner diameter); 11) stepper motor (NEMA 17); 12) b) cross section of the reactor: connector for stir plate control; 13) light source (150 W Hg medium-pressure lamp); 14) mount for the central UV filter reservoir; 15) microcontroller unit (MCU), Arduino Uno with CNC shield and stepper driver. c) Photograph of the reactor system.

The main components of the reactor are shown in Fig. 1. A centrally positioned cooling jacket (1) is enclosed within an annular reservoir intended to contain long-pass filter solutions, providing an effective optical path length of 30 mm (3). The annular reservoir is positioned concentrically within a custom-designed 3D-printed support housing (14), ensuring precise alignment. The light source (13) is placed inside the cooling jacket; in the configuration used here, a 150 W medium-pressure mercury lamp (TQ150, Peschl Ultraviolet GmbH) was employed. Light sources with similar cylindrical irradiation geometries can also be used without requiring modification to the reactor design.

**Fig. 2:**
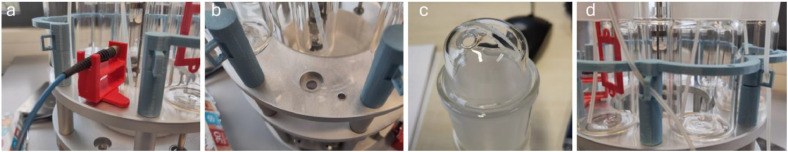
| a) spectrometer optic (PLA) b) alignment drilling for the spectrometer optic c) Frame slit and sampling hole of the sample vessels d) vessel holder with eyelets for guiding the sampling capillary.

Six irradiation vessels (2) are symmetrically arranged in vessel holders (4) around the central reservoir on the vessel support plate. The vessels are custom fabricated with a slot for suspending photocatalytic membranes and a top opening that allows automated or manual sampling. As shown in Fig. 2(c), the upper section of the vessel is designed to minimize sample evaporation while allowing insertion of membrane holders and a sampling capillary. To facilitate stable positioning of the sampling line, a small sampling capillary guide extends from the vessel holder, as shown in Figure 2(d), supporting and aligning the sampling tube outside the irradiation vessel.

In addition to the six irradiation vessels, a seventh position on the vessel support plate (Fig. 2a) is designed for the integration of spectrometer optics (5) (sensoTEC USB 5000). This configuration enables *in situ* measurement of the lamp emission spectrum downstream of the optical filter reservoir. Precise alignment of the spectrometer with the lamp axis is ensured by a dedicated bore aligned with the lamp centre (Fig. 2b).

Mixing within each irradiation vessel is achieved using magnetic stir bars (6), actuated by bottom-mounted rotating shafts with magnet heads. Each shaft is supported by two ball bearings and fixed in place using shaft collars (10). Stable magnetic coupling is ensured by positioning a thin acrylic plate above the stir bars. All six shafts are driven simultaneously by a stepper motor (Nema 17 Standard) (11) via GT2 belts (172 mm length, 6 mm wide) and pulleys (GT2, 21 teeth). Stepper motor operation and rotational speed are regulated by a microcontroller unit (15) comprising an Arduino Uno equipped with a CNC shield and a stepper driver. Control and adjustment are performed using an external control box (12) fitted with a rotary encoder. The detailed wiring schematic of the reactor is provided in the supplementary information (Figure S1).

**Fig. 3:**
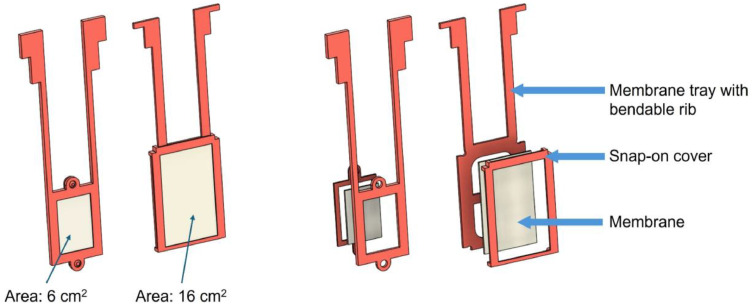
| 3D models of two photocatalytic membrane holder assemblies for the photocatalytic setup — assembled view (left) and exploded view (right).

For experiments involving immobilized photocatalysts, TiO_2_-decorated PES membranes [5,6], were mounted in custom-fabricated 3D-printed membrane holders. The holders were designed to align membrane areas of 5 and 16 cm² toward the light source. The corresponding configurations are shown in Fig. 3.

The photocatalytic membranes are retained within the holders by a snap-fit mechanism. Each membrane holder incorporates a flexible rib with two discrete positions, allowing adjustment of the membrane position relative to the light source. The membrane holders, spectrometer optic holder, and vessel holders were fabricated from polylactic acid (PLA) using a desktop fused deposition modelling (FDM) 3D printer (Elegoo Neptune 3), enabling flexible integration of the membrane holders within the vessels. Detailed blueprints are provided in the supplementary information (Section S7).

### Phase 2: reactor characterisation: optical properties

#### Spectral control

**Fig. 4:**
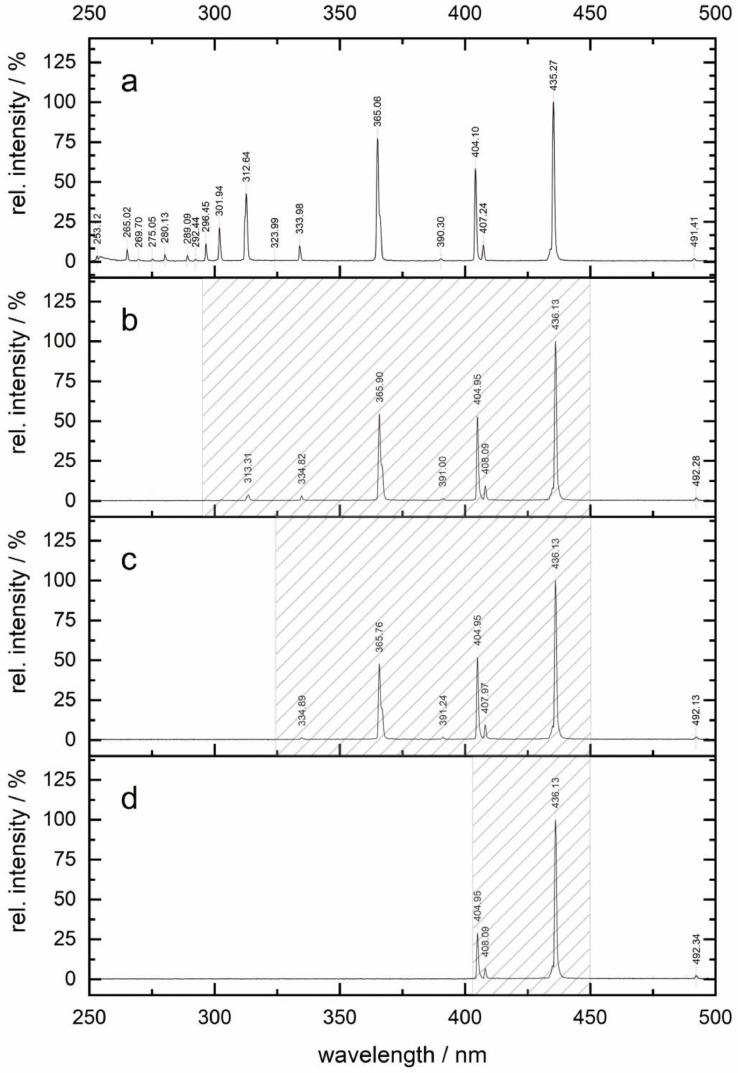
| Emission spectra of a 150 W mercury medium-pressure lamp, normalized to the maximum intensity (100 %), under different filter conditions: a) no filter, b) borosilicate glass (cut-off at λ=295 nm), c) nitrate solution (cut-off at λ=325 nm), d) nitrite solution (cut-off at λ=405 nm). Hatched area shows the gap which was characterized by chemical actinometry.

The PMSR incorporates a design feature of the MAPMR that employs UV long-pass filter solutions for selective investigation of photocatalytic processes under a white light source. Filter solutions previously reported for the MAPMR were used and introduced into the central reservoir [4]. In the present configuration, the filter solutions are integrated into the central UV long-pass filter reservoir, allowing rapid exchange of the filter solutions, which reduces cleaning efforts compared to the earlier design. The central reservoir is fabricated from borosilicate glass with a wall thickness of 2 mm. For the experiments, it was filled with either potassium nitrate (25 g/L) or sodium nitrite (25 g/L) solutions to selectively attenuate specific regions of the lamp emission spectrum. The resulting spectra are shown in Fig. 4. This approach enables selective modification of the lamp emission spectrum without changing the light source. For experiments requiring full-spectrum irradiation, the central reservoir can be removed from the reactor.

As shown in Fig. 4, both the filter solutions and the borosilicate glass contributed to the attenuation of ultraviolet wavelengths. In previous studies, including our own [4], potassium hydrogen phthalate has commonly been used to obtain a sharp cut-off at wavelengths below 295 nm. In the present reactor configuration, however, the use of potassium phthalate is not required, as the borosilicate glass provides sufficient attenuation in this spectral region, as shown in Fig. 4b [4,7].

#### Optical path and light field geometry characterization

**Fig. 5:**
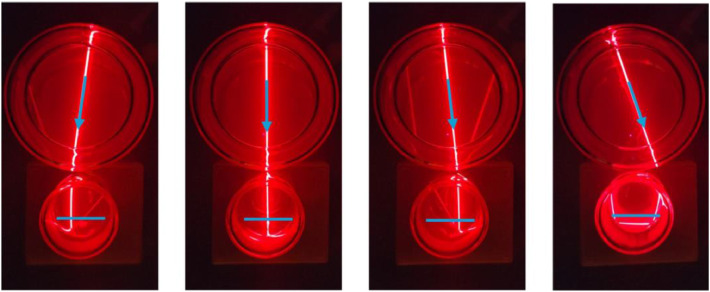
| Visualization of the optical beam path using a 1- mW, 650- nm laser pointer and a 5 wt % LUDOX (colloidal silica) suspension. Central filter vessel (top), irradiation vessel (bottom). Blue arrows indicate the irradiation direction, and the blue bars indicate photocatalytic membranes in the irradiation vessel.

To evaluate the optical properties of the PMSR, a separate light-path visualization experiment was conducted. The central filter reservoir and one irradiation vessel were filled with a 5 wt % colloidal silica suspension, prepared by diluting LUDOX AS-40 colloidal silica (40 wt %). The components were arranged to replicate the geometry used in the reactor (Fig. 5). A laser pointer (650 nm, 1 mW) was aligned with the central reservoir such that the beam passed through the centre of the filter reservoir, and the beam position was incrementally adjusted until it no longer intersected the irradiation vessel. Representative beam positions are shown in Fig. 5.

The results (Fig. 5) indicate that the beam passed through the irradiation vessel as approximately parallel rays over a range of angular positions, despite the radial emission geometry of the central lamp source. Based on this observation, the incident light within the reactor can be considered approximately quasi-collimated at the membrane surface, where the membranes are oriented perpendicular to the beam direction. A decrease in the local light intensity toward the membrane edges is expected due to the increased optical path length relative to the membrane centre. In the far-right panel of Fig. 5, total internal reflection of the laser beam is observed at the glass–air interface as a result of the refractive-index contrast, resulting in an apparent discontinuity of the beam. Avoidance of total internal reflection would require modification of the irradiation vessel cross-section, for example by using a rectangular shape; however, this would require a more precise alignment of the irradiation vessels, whereas round-shaped vessels avoid this adjustment step.

#### Determination of the photon flux by chemical actinometry

To characterize the light properties within the reactor system under the applied UV long-pass filter conditions, the photon flux was determined by photolytic degradation of potassium tris(oxalato)ferrate(III), also known as ferrioxalate [8]. Photon fluxes were measured separately for each filter solution. For unfiltered medium-pressure mercury lamp emission, the photon flux was calculated independently due to the rapid photodegradation of ferrioxalate under UVC irradiation. Details of the calculation procedure, together with the full medium-pressure lamp emission spectrum from 200 to 700 nm, are provided in the supplementary information (Section S4).

For accurate determination of the photon flux, the spectral emission profile of the lamp f(λ), was recorded over the selected wavelength range and normalized to a unit area of 1. The wavelength-dependent molar absorption coefficient, ε(λ), was determined experimentally, while the corresponding quantum yield, φ(λ), was taken from the literature [9]. The experimentally determined molar absorption coefficients in the wavelength range of 250–450 nm are presented in the supporting information (Section S4) and are consistent with the reported literature values [10]. The photon flux ($F_{p}$) was calculated using an equation adapted from Canonica et al [11], accounting for the wavelength dependence of the ferrioxalate quantum yield (Eq.1). Three long-pass filter solutions with different cut-off wavelengths were applied.

(1) $$\begin{array}{c} F_{p}\left(\lambda =\mathrm{cut}-\mathrm{off}\;\mathrm{wave}\mathrm{leng}\mathrm{th}-450\;\mathrm{nm}\right)=\frac{k_{\mathrm{obs},\;\mathrm{Ferr}\mathrm{ioxa}\mathrm{late}}}{2.303\;\sum_{\mathrm{cut}-\mathrm{off}\;\mathrm{wave}\mathrm{leng}\mathrm{th}}^{450}\left(f_{p,\lambda }\varepsilon_{\lambda }\phi_{\lambda }\right)} \end{array}$$

**Fig. 6:**
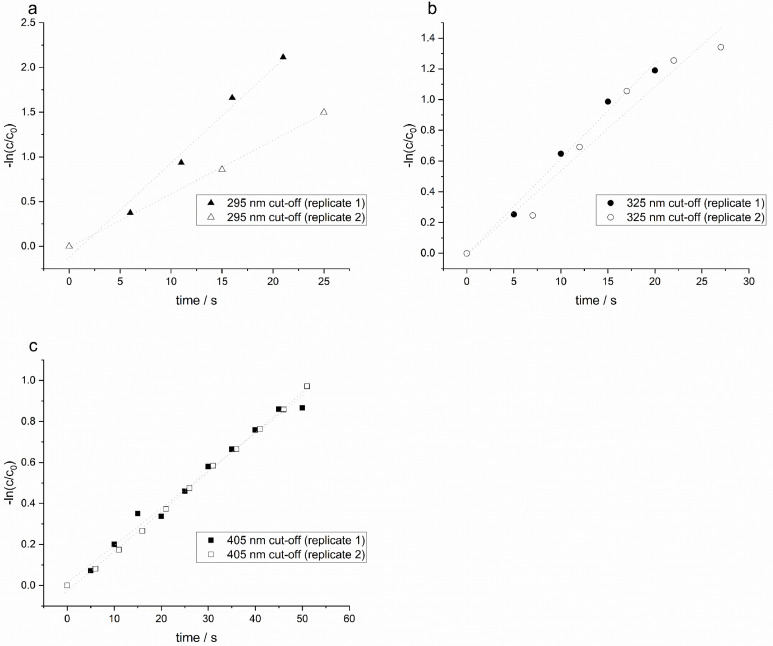
| Linearized degradation data [-ln(c/c_0_) vs time] of ferrioxalate measured as ferroin complex applying three light filters:(a) borosilicate glass (λ=295 nm cut-off), (b) nitrate (λ=325 nm cut-off), (c) nitrite (λ=405 nm cut-off). Note: c_0_ and c denote the concentrations at *t* = 0 and at any given time t during the reaction, respectively.

The determination and derivation of the individual parameters in Eq.1 are described below. To determine k_obs_, a 300 µM potassium ferrioxalate solution was introduced into the irradiation vessel. After a 10 min warm-up period, medium-pressure mercury lamp was inserted into the cooling jacket of the PMSR, defining time zero. During irradiation, the reaction mixture was continuously withdrawn from the irradiation vessel using a peristaltic pump, while the PMSR was fully wrapped with aluminium foil to prevent interference from external light. During the experiment, 1.7 mL of the aliquots were withdrawn at 5 s intervals. Subsequently, 1.5 mL of each aliquot was mixed with 0.75 mL of acidified 1 M sodium acetate buffer (acidified with 0.5 M H_2_SO_4_), and 2.25 mL of a 0.1 M 1,10-phenanthroline solution. The resulting mixtures were allowed to react for at least 30 min in the dark to ensure complete complexation. Under acidic conditions, 1,10-phenanthroline forms a stable ferroin complex with Fe(II), which is produced during the photoreduction of Fe(III) in the ferrioxalate complex. The absorbance of the resulting ferroin complex was measured at 510 nm using UV2600i spectrophotometer (Shimadzu, Germany). Quantification was performed using a calibration curve prepared from ferrous (II) sulphate standards following the same complexation procedure. The calibration curve is provided in the supporting information (Section S5; Figure S4). The degradation kinetics of potassium ferrioxalate is shown in Fig. 6.

**Fig. 7:**
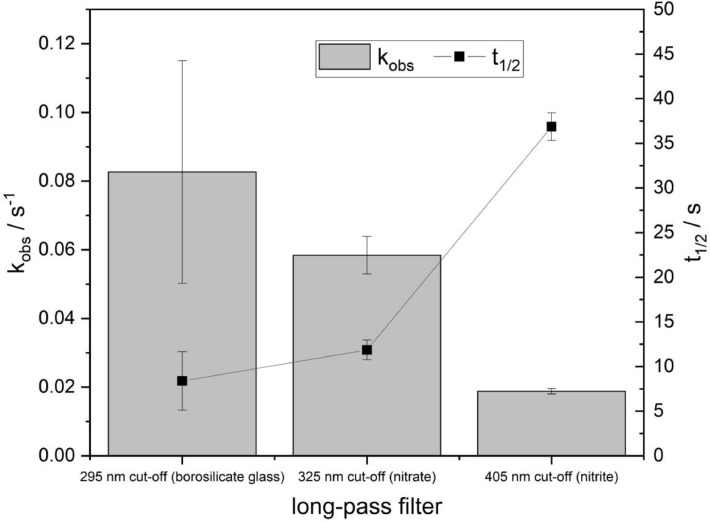
| Pseudo-first-order degradation constants of potassium tris(oxalato)ferrate(III) (ferrioxalate) at three filter settings. Borosilicate glass (λ=295 nm cut-off), nitrate (λ=325 nm cut-off), nitrite (λ=405 nm cut-off).

Fig. 6 shows the pseudo-first order rate kinetics of ferrioxalate degradation determined under three different filter conditions. For slower reactions, sampling over the extended time of 60 s was performed because concentration changes occur more gradually, resulting in improved measurement accuracy and reproducibility. To enable comparison of the ferrioxalate photoreaction rate constants, the slopes of the corresponding linear regressions were used to calculate pseudo-first-order rate constants and half-lives. The resulting values for the different filter conditions are compared in Fig. 7.

The data exhibit a consistent trend with the applied long-pass filter conditions, with higher k values obtained for lower wavelength cut-off values, corresponding to a higher number of photons available in the reaction solution. Since ferrioxalate is highly sensitive to UV irradiation and the experimental exposure times were limited to only a few seconds, increased standard deviations were observed under conditions employing lower wavelength cut-off filters.

For calculation of the photon flux, it is necessary to define the effective spectral range of the dataset from the cut-off wavelength up to 450 nm, corresponding to the absorption maximum of ferrioxalate. This approach follows Kasha’s rule, which assumes that direct photolysis does not occur in the absence of photon absorption by the probe compound [12]. Thereby, $f_{j}$ represents a relative single data point within the lamp emission spectrum over the investigated wavelength range, j indexes the equidistant wavelength data points of Δλ = 1 nm and a corresponding intensity, divided by the sum of intensities within the investigated wavelength region. The sum of all $f_{j}$ values equal one for each chosen filter, which is required to correctly determine the photon flux for the respective long-pass filter based on Eq. (1). The following two equations, Eq. 2 and Eq. 3, show how $f_{j}$ can be calculated.

(2) $$f_{j}=\frac{I_{j}}{\sum_{j\in cut-off\;wavelength-450\;nm}I_{j}}$$

(3) $$\sum_{j\in cut-off\;wavelength-450\;nm}f_{j}=\;\frac{\sum_{j\in cut-off\;wavelength-450\;nm}I_{j}}{\sum_{j\in cut-off\;wavelength-450\;nm}I_{j}}\;=1$$

Accurate determination of the photon flux requires knowledge of the wavelength-dependent quantum yield of ferrioxalate over the investigated spectral range. Literature values for ferrioxalate quantum yields are available for the wavelengths between 350 and 450 nm [9]. By combining experimental measurements with the published data, Demas et al [9] proposed a linear relationship describing the quantum yield as a function of wavelength:

(4) Φ(λ)=−0.0037λ+2.602

This relationship was used to estimate the quantum yield over the wavelength range of 250–450 nm and subsequently used to calculate the photon flux according to Eq. (1). It should be noted that this approach introduces uncertainty at wavelengths below 350 nm, as this spectral region was not investigated by Demas et al [9].

To determine not only the photon flux but also the corresponding emitted energy for a given filter setting, the mean wavelength of the respective light spectrum must be calculated (as exemplarily described in the section S4; Eq. S1 ongoing). In combination with Eq. 1, this enables estimation of both the photon flux and the emitted energy. The resulting focus wavelengths are summarized in Table 1.

**Table 1:** Focus wavelength within the tested spectral area with the respective UV-filter from the cut-off until 450 nm.

Spectral region	Focus wavelength $\lambda_{focus}$ [nm]
No filter (spectrum form 200 until 700 nm)	431
Borosilicate glass (295 nm cut-off)	404
Nitrate (325 nm cut-off)	406
Nitrite (405 nm cut-off)	438

Using the focus wavelength of the spectral range, the corresponding photon energy was calculated, enabling determination of the energy flux ($E_{flux}$) according to Eq. (5), with the constants h (Planck constant), c (speed of light), and $N_{A}\;$(Avogadro constant):

(5) $$E_{flux}=\;F_{p}\;N_{A}\;E_{Photon}=\;F_{p}\;N_{A}\;\frac{h\;c}{\lambda_{focus}}\left[W\;m^{-2}\right]$$

The theoretical energy flux values and the actinometrically determined photon flux values are summarized in Table 2, defining the PMSR light properties with the medium-pressure mercury lamp.

**Table 2:** Photon flux of the photocatalysis setup under the respective filter settings.

Spectrum	Photon flux (µEinstein/m^2^/s)	Energy flux (W/m^2^/s)
Without filter (theoretically calculated in SI, full spectrum: ∼200- 700 nm)	982	273
borosilicate glass (295–450 nm)	851±334	252±99
nitrate (325–450 nm)	763±71	225±21
nitrite (405–450 nm)	362±49	99±13

## Method validation

The validation stage was conducted through photocatalytic degradation experiments to assess the overall performance of the PMSR under well-controlled conditions. All experiments were carried out under identical operational parameters to ensure consistency and reproducibility. Particular emphasis was placed on comparing the efficiencies of immobilized and suspended photocatalyst systems under identical conditions.

### Phase 3: photocatalytic treatment

As a proof of concept, the PMSR was used to investigate the photo(catalytic) degradation of the common antibiotic amoxicillin (AMX) using TiO_2_ decorated PES membranes [6]. The synthesis procedure for these membranes has been described previously [6]. To allow direct comparison between immobilized and suspended photocatalyst configurations, experiments were performed using equivalent amounts of photocatalyst in membrane-supported and suspension-based systems. Photocatalytic membrane performance was evaluated against TiO_2_ particles in suspension at a concentration of 124 mg/L (corresponding to a total mass of 14 mg per vessel), using a TiO_2_-decorated PES membrane area of 16 cm^2^ containing an equivalent photocatalyst mass (14 mg). Therefore, each irradiation vessel was filled with 110 mL of a 50 µM AMX solution. The solution pH was adjusted to 7 using a 5 mM phosphate buffer, with fine adjustment performed using NaOH or phosphoric acid. Membrane samples were prepared by cutting the TiO_2_-decorated PES membranes slightly larger than 16 cm^2^ to allow secure fixation within the membrane trays. The membranes were immersed in the reaction solution and positioned vertically facing the light source. Magnetic stirring was applied in all vessels to ensure homogeneous mixing. Silicone tubing (length 1 m, inner diameter 2 mm) was inserted into the designated openings of the vessel lids to facilitate the sampling.

**Fig. 8:**
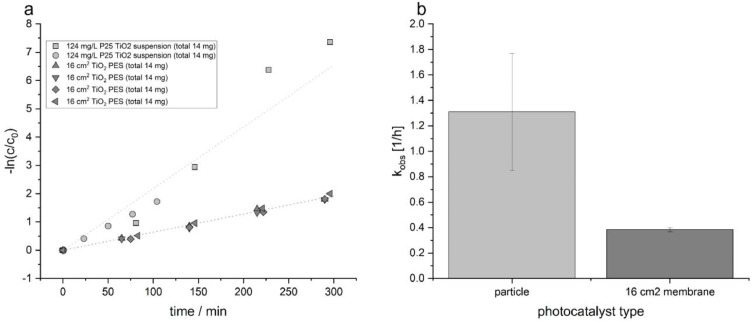
| a) Linearized degradation data [-ln(c/c_0_) vs time] for AMX (initial concentration 50 µM), comparing a TiO_2_ particle suspension (124 mg/L; total TiO_2_ = 14 mg in 110 mL) with a TiO_2_-modified polyethersulfone (PES) membrane (membrane area = 16 cm²), irradiated with a 25 g/L nitrate filter (λ=325 nm cut-off). b) Apparent rate constants (k_obs_), reported as absolute values.

For these experiments, a 25 g/L potassium nitrate solution was used as a UV cut-off filter in the central filter reservoir (Fig. 4c). The entire reactor assembly was wrapped in aluminium foil to stabilize irradiation conditions and minimize photon losses. Samples were irradiated for up to 300 min. Aliquots were withdrawn using a peristaltic pump, the first millilitre was discarded to avoid dead-volume effects, after which the sample was collected and the sampling capillaries were flushed backwards. Each sampling event required approximately 1 min. The concentration of AMX in the samples was quantified by LC-MS as reported recently [4]. The corresponding degradation results are presented in Fig. 8.

Compared to the membrane-supported configuration, the TiO_2_ particle suspension exhibited a 3.4-fold higher AMX degradation rate. The membrane system, however, showed highly reproducible performance with consistent degradation rates across repeated experiments. Notably, the membrane occupies a substantially smaller effective reactor volume of approximately 8 mL (16 cm² × 0.5 cm), compared to 110 mL for the particle suspension, corresponding to a membrane-to-suspension volume ratio of 1:13.75. Consequently, although the immobilized system shows a 3.4-fold lower degradation rate, it requires 13.75-fold less volume, underscoring the practical advantages of membrane-based operation, including compact reactor design, facilitated catalyst reuse, and the potential for simultaneous filtration.

**Fig. 9:**
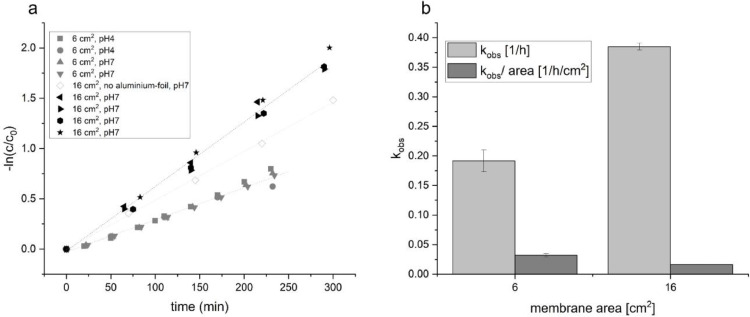
| a) Linearized degradation data [-ln(c/c_0_) vs time] for AMX (initial concentration 50 µM) obtained using a TiO_2_-modified polyethersulfone (PES) membrane with a 25 g/L nitrate filter (λ=325 nm cut-off) at two membrane surface areas. b) Apparent rate constants (k_obs_), reported as absolute values and normalized by membrane area.

Under the employed filter configuration, no substantial AMX degradation was observed from direct photolysis using either borosilicate glass, nitrate, or nitrite filters, nor from sorption or hydrolysis. The corresponding reference and control experiments are presented in the supporting information (Figure S5). Subsequently, the effects of (i) reducing the active membrane area to 6 cm^2^; (ii) operating the reactor without aluminium foil wrapping; and (iii) conducting experiments under acidic conditions (pH 4) were investigated to further evaluate the influence of reactor configuration and operating parameters on process performance (Fig. 9).

The data show that no measurable difference was observed between the experiments conducted at pH 4 and pH 7; therefore, the pH 4 and pH 7 datasets for the 6 cm² membrane are combined in panel b (Fig. 9). Wrapping the reactor with aluminium foil decreases the degradation rate by approximately 24 % (to 0.76 of the reference), likely due to reflective losses that reduce the photon flux in the solution. In panel b, k_obs_ values (excluding the no-foil experiment) are summarized and normalized to membrane area. Under this normalization, the trend reverses and the smaller membrane performs slightly better, which is attributed to the reactor’s illumination geometry (see Fig. 5), with the highest irradiance concentrated at the membrane centre. In absolute terms, the 16 cm² membrane achieves the highest overall conversion.

In conclusion, in this study a versatile reactor design was developed to enable both systematic screening of the degradation conditions and detailed evaluation of the photocatalytic materials. The system addresses key limitations of conventional merry-go-around photoreactors by allowing the use of immobilized photocatalysts under well-controlled and efficiently mixed batch conditions. A key feature of the design is the integration of spectral long-pass filters, which enables selective investigation of photo(catalytic) pathways under defined irradiation regimes. Reactor characterization, including path beam analysis, irradiation distribution, and photon flux determination, provides a reliable basis for quantitative assessment of photocatalytic degradation processes. Besides, the reactor allows targeted analysis of the transformation products, supporting mechanistic insights and facilitating the optimization of photocatalytic performance.

## Limitations

• The emission spectrum of the irradiation source is non-uniform, requiring precise characterization and, depending on the wavelength range, the use of appropriate chemical or electronic actinometers.
• The lack of suitable optical filters limits selective wavelength control, as catalyst excitation and substrate absorption may overlap (e.g., TiO₂ activation in the UVA region).
• Reaction intermediates and matrix components may absorb incident light, attenuating photon flux to the catalyst and reducing control over irradiation conditions.

## Ethics statements

None.

## CRediT author statement

**Michael S. Leupold**: Conceptualization, Methodology, Investigation, Visualization, Writing – original draft, Writing – review & editing. **Klaus Kerpen:** Conceptualization – review & editing. **Anam Asghar:** Writing – review & editing. **Lukas Fischer**: Writing – review & editing. **Torsten C. Schmidt:** Funding acquisition, Writing – review & editing.

## Declaration of generative AI and AI-assisted technologies in the writing process

During the preparation of this work, ChatGPT 5.4 was used as an AI-assisted technology via the University of Duisburg-Essen AI portal (https://www.uni-due.de/de/digitalisierung/ki-portal/) in order to verify grammar and spelling. After using the tool, the authors reviewed and edited the content as needed and take full responsibility for the content of the published article.

## Declaration of competing interest

The authors declare that they have no known competing financial interests or personal relationships that could have appeared to influence the work reported in this paper.

## Data Availability

Data will be made available on request.

## Appendix Supplementary materials

Image, application 1
