# Supplementary material for: Development of a photocatalytic membrane screening reactor (PMSR) for standardized evaluation of immobilized photocatalytic support materials
Source: MethodsX. 2026 May 2;16:103938. doi: 10.1016/j.mex.2026.103938 (PMC13191272; doi:10.1016/j.mex.2026.103938)
Supplement: Supplementary file 1 [file mmc1.docx]

**Development of a Photocatalytic Membrane Screening Reactor (PMSR) for Standardized Evaluation of Immobilized Photocatalytic Support Materials**

Michael S. Leupold^a, b^, Anam Asghar^a, b*^, Klaus Kerpen^a^, Lukas Fischer^b, c^, Torsten C. Schmidt^a, b, d^

*^a^Instrumental Analytical Chemistry, University of Duisburg-Essen, Universitätsstr.5, Essen 45141, Germany.*

*^b^Centre for Water and Environmental Research (ZWU), University of Duisburg-Essen, Universitätsstr.2, Essen 45141, Germany.*

*^c^Technical Chemistry II, University of Duisburg-Essen, Universitätsstr.5, Essen 45141, Germany.*

*^d^IWW Water Center, Moritzstr.26, Mülheim an der Ruhr 45476, Germany.*

Supporting Information

**Corresponding author**

| **Subject area** | Chemistry |
| --- | --- |

Anam Asghar

Email: [anam.asghar@uni-due.de](mailto:anam.asghar@uni-due.de)

Tel. +49 201 183-6778

Fax. +49 201 183-6773

# Supplementary material *and/or* additional information

## S1 List of chemicals:

*Table 1 | List of chemicals used in this study.*

| **Compound** | **Formula** | **Purity** | **Manufacturer** |
| --- | --- | --- | --- |
| 1, 10-Phenantrolin-Monohydrate | C_12_H_8_N_2_ H_2_O | ≥ 99,0%, p.a. | SIGMA ALDRICH CHEMIE GmbH, Riedstr. 2, 89555 Steinheim, Germany |
| Amoxicillin | C_16_H_19_N_3_O_5_S | 95,0-102,0%, anhydrous basis | SIGMA-ALDRICH, Co., 3050 Spruce Street, St. Louis, MO 63103 USA |
| Disodiumhydrogen-phosphate-Dehydrate | Na_2_HPO_4_ 2 H_2_O | ≥ 99,5%, p.a. | Carl Roth GmbH + Co. KG, Schoemperlenstr. 3-5, 76185 Karlsruhe, Germany |
| Fe(II)-sulfate-Heptahydrate | FeSO_4_ 7 H_2_O | ≥ 99,0%, p.a., ACS | SIGMA ALDRICH CHEMIE GmbH, Riedstr. 2, 89555 Steinheim, Germany |
| Potassium hydrogen-phthalate (KHP) | C_8_H_5_KO_4_ | ≥ 99,5%, p.a. | Merck KGaA, Frankfurter Str. 250, 64271 Darmstadt, Germany |
| Potassium nitrate | KNO_3_ | ≥ 99%, p.a. | Merck KGaA, Frankfurter Str. 250, 64271 Darmstadt, Germany |
| Potassium trioxalato-ferrat(III)-Trihydrate | K3Fe(C2O4)3 ∙3 H2O | n.a. | ThermoFisher GmbH, Erlenbachweg 2, 76870 Kandel, Germany |
| LUDOX® HS-40 colloidal Silicone dioxide | SiO_2_ | 40 wt. % suspension in H2O | SIGMA ALDRICH CHEMIE GmbH, Riedstr. 2, 89555 Steinheim, Germany |
| Sodium acetate | C_2_H_2_O_2_Na | ≥ 99%, p.a. | Merck KGaA, Frankfurter Str. 250, 64271 Darmstadt, Germany |
| Sodium dihydrogen-phosphate-Dehydrate | NaH2PO4 ∙2 H2O | extra pure | Riedel-de Haen, Wunstorfer Str. 40, 30926 Seelze, Germany |
| Sodium hydroxide | NaOH | 1 M | Bernd Kraft GmbH, Stempelstraße 6, 47167 Duisburg, Germany |
| Sodium nitrite | NaNO_2_ | 99,9% | Merck KGaA, Frankfurter Str. 250, 64271 Darmstadt, Germany |
| Phosphoric acid | H_3_PO_4_ | ≥ 85 wt. % in H_2_O | SIGMA ALDRICH CHEMIE GmbH, Riedstr. 2, 89555 Steinheim, Germany |
| Hydrochloric acid | HCl | 37%, Reag. Ph. Eur. | VWR International S.A.S., Rue d'Aurion 1-3, 93114, France |
| Sulphuric acid | H_2_SO_4_ | 95% | VWR International S.A.S., Rue d'Aurion 1-3, 93114, France |

## S2 Electronic setup

The reactor stirring system was controlled using a microcontroller-based setup. A microcontroller unit (MCU; Arduino Uno) was interfaced with a stepper motor driver module (A4988-compatible), which controlled a NEMA 17 stepper motor. Motor power was provided via an external 24 V DC power supply. User input was implemented through a rotary encoder connected to the MCU digital input pins, allowing adjustment of the motor rotational speed. The encoder signals were processed by the microcontroller, which generated corresponding step and direction signals for the motor driver. A detailed wiring schematic, including pin assignments and power connections, is provided in the Figure S1.


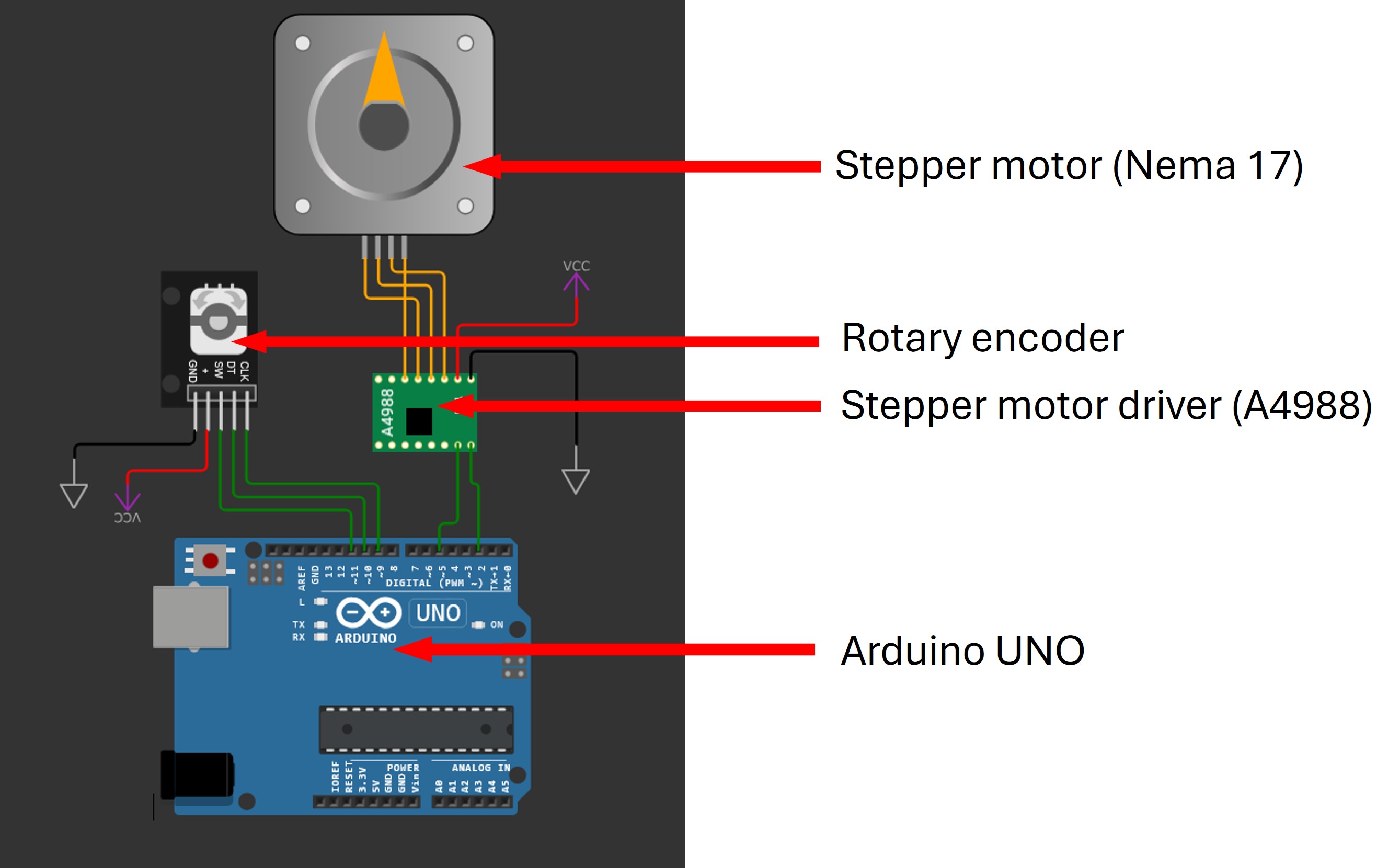


Figure S1 | Schematic of the electronic used in the screening reactor.

## S3 C/C++ code of the electronics

#include <Encoder.h>

#define STEP_PIN 2

#define DIR_PIN 5

#define ENCODER_CLK_PIN 9

#define ENCODER_DT_PIN 10

#define ENCODER_BTN_PIN 11

Encoder myEnc(ENCODER_CLK_PIN, ENCODER_DT_PIN);

bool motorOn = false;

long oldPosition = 0;

int delayTime = 2000; // Initial speed: 2000 microseconds

void setup() {

pinMode(STEP_PIN, OUTPUT);

pinMode(DIR_PIN, OUTPUT);

pinMode(ENCODER_BTN_PIN, INPUT_PULLUP); // Enable internal pull-up resistor

Serial.begin(9600); // Start serial communication

digitalWrite(DIR_PIN, HIGH); // Set rotation direction

}

void loop() {

long newPosition = myEnc.read() / 4;

// Divide encoder value by 4 (increase sensitivity)

if (newPosition != oldPosition) {

long change = newPosition - oldPosition;

oldPosition = newPosition;

delayTime -= change * 50; // 50 microseconds per encoder step

delayTime = constrain(delayTime, 50, 2000);

// Limit delay between 50 and 2000 microseconds

Serial.print("Current delay time: ");

Serial.print(delayTime);

Serial.println(" microseconds");

}

if (digitalRead(ENCODER_BTN_PIN) == LOW) {

motorOn = !motorOn;

delay(300); // Button debounce delay

}

if (motorOn) {

digitalWrite(STEP_PIN, HIGH);

delayMicroseconds(delayTime);

digitalWrite(STEP_PIN, LOW);

delayMicroseconds(delayTime);

}

}

## S4 Theoretical calculation of the photon flux full mercury medium pressure lamp spectrum

For the theoretical calculation of the photon flux, the medium-pressure mercury lamp was assumed as radiation source emitting photons along radial directions. The lamp power was set to 150 W, with the assumption that 10% of the electrical input was converted into photon energy.

The total photon power ($P_{photons})$ was calculated from the medium-pressure mercury lamp power ($P_{lamp})$ using a reduction factor f:

| $P_{photons}=P_{lamp} f$ | Eq. S1 |
| --- | --- |

| $P_{photons}=150 W 0.10=15 W$ | Eq. S2 |
| --- | --- |

Based on the surface of a sphere with a radius corresponding to the distance from the lamp centre to the membrane, the spherical surface area was calculated as:

| $A_{sphere}=4\pi r^{2}=4\pi{(0.065 m)}^{2}=4\pi\cdot0.004225=0.0531 m^{2}$ | Eq. S3 |
| --- | --- |

The mean wavelength was subsequently determined by calculating the centre of the full lamp emission spectrum along the wavelength axis (as indicated in Figure S2), yielding:

| $\lambda_{focus}=\frac{\int\lambda I\left( \lambda\right)d\lambda}{\int I(\lambda\boldsymbol{)}d\lambda}=\frac{\sum_{i} \lambda_{i}I_{i}}{\sum_{i} I_{i}}=431 nm$ | Eq. S4 |
| --- | --- |


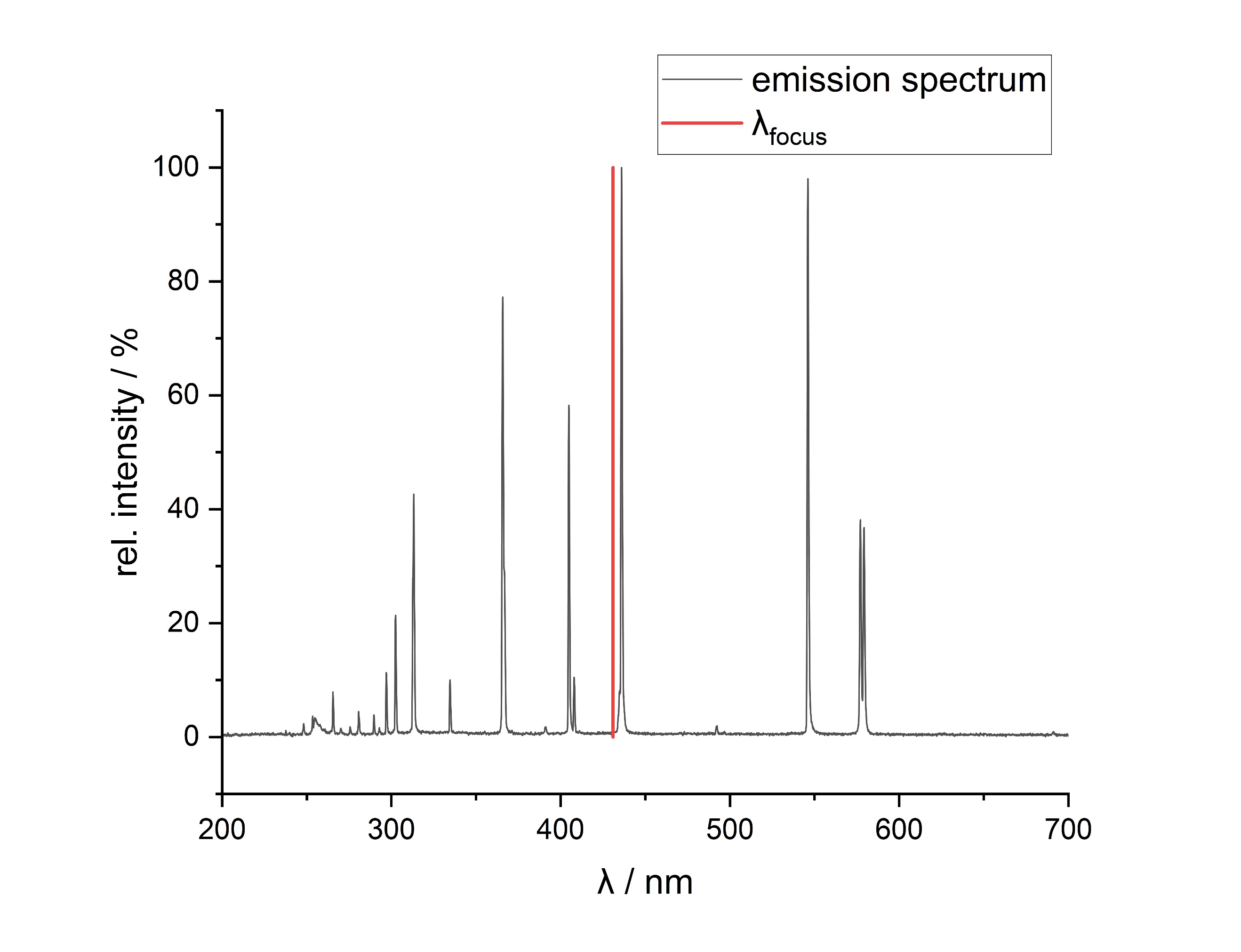


Figure S2 | full mercury medium-pressure lamp spectrum with indicated $\lambda_{center}$in red.

Subsequently, the energy of a single photon ($E_{photon}$) with the $\lambda_{\mathrm{centre}}$ was calculated as:

| $E_{photon}=\frac{hc}{\lambda_{focus}}= \frac{6.626*{10}^{-34}Js*2.998*{10}^{8}\frac{m}{s}}{431*{10}^{-9} m}=4.61*{10}^{-19}\frac{J}{photon} (2.88 eV)$ | Eq. S5 |
| --- | --- |

Based on the energy of a single photon, both the photon fluence and the energy flux were calculated.

| $E_{flux}\left( r \right)=\frac{P_{lamp}*f}{E_{photon}* 4\pi r^{2}}$ $\left[ photons m^{-2}s^{-1} \right]$ | Eq. S6 |
| --- | --- |
| $E_{flux}=\frac{150\frac{J}{s}*0.10}{4.61*{10}^{-19}J* 4\pi{(0.065 m)}^{2}}=6.13*{10}^{20}\frac{photons}{m^{2}s}=982\frac{\mu Einstein}{m^{2} s}$ | Eq. S7 |
| $E\left( r \right)=272\frac{W}{m^{2}}=27.2\frac{mW}{{cm}^{2}}$ | Eq.S8 |

## S5 Determination of the molar absorption coefficient of ferrioxalate

The molar absorption coefficients of ferrioxalate are available in the literature, for example in Fonseca et al. (2003) [1]. Nevertheless, the coefficients were determined experimentally over smaller wavelength intervals in this study. For this purpose, a 2 mM stock solution of potassium trioxalatoferrate (III) in water (acidified to pH 2 with sulfuric acid) was prepared and diluted in the dark to the concentrations of 50, 100, 200, 300, and 500 µM. The absorption profiles were recorded using UV-vis spectrometer (Shimdazu UV2600i). The absorption spectra are shown in Figure S3(a). The molar absorption coefficient at each wavelength was calculated from the slope according to the Lambert–Beer law. The coefficient of determination (R^2^) from the linear regression was greater than 0.99 across the wavelength range of 250-441 nm. The resulting molar absorption coefficients within this range are shown in Figure S3(b).


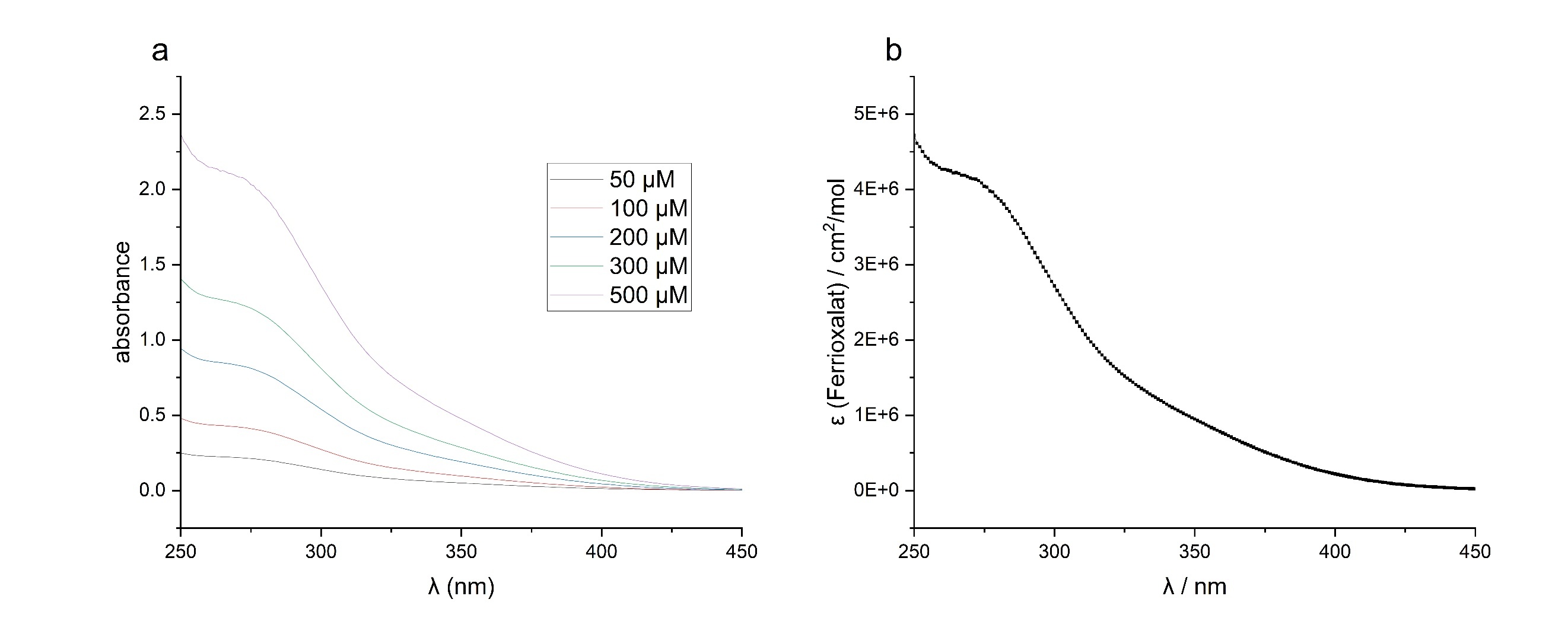


Figure S3 | a) Absorption spectra of potassium trioxalatoferrate(III) (ferrioxalate) at different concentrations. b) Calculated molar extinction coefficients of potassium trioxalatoferrate(III).


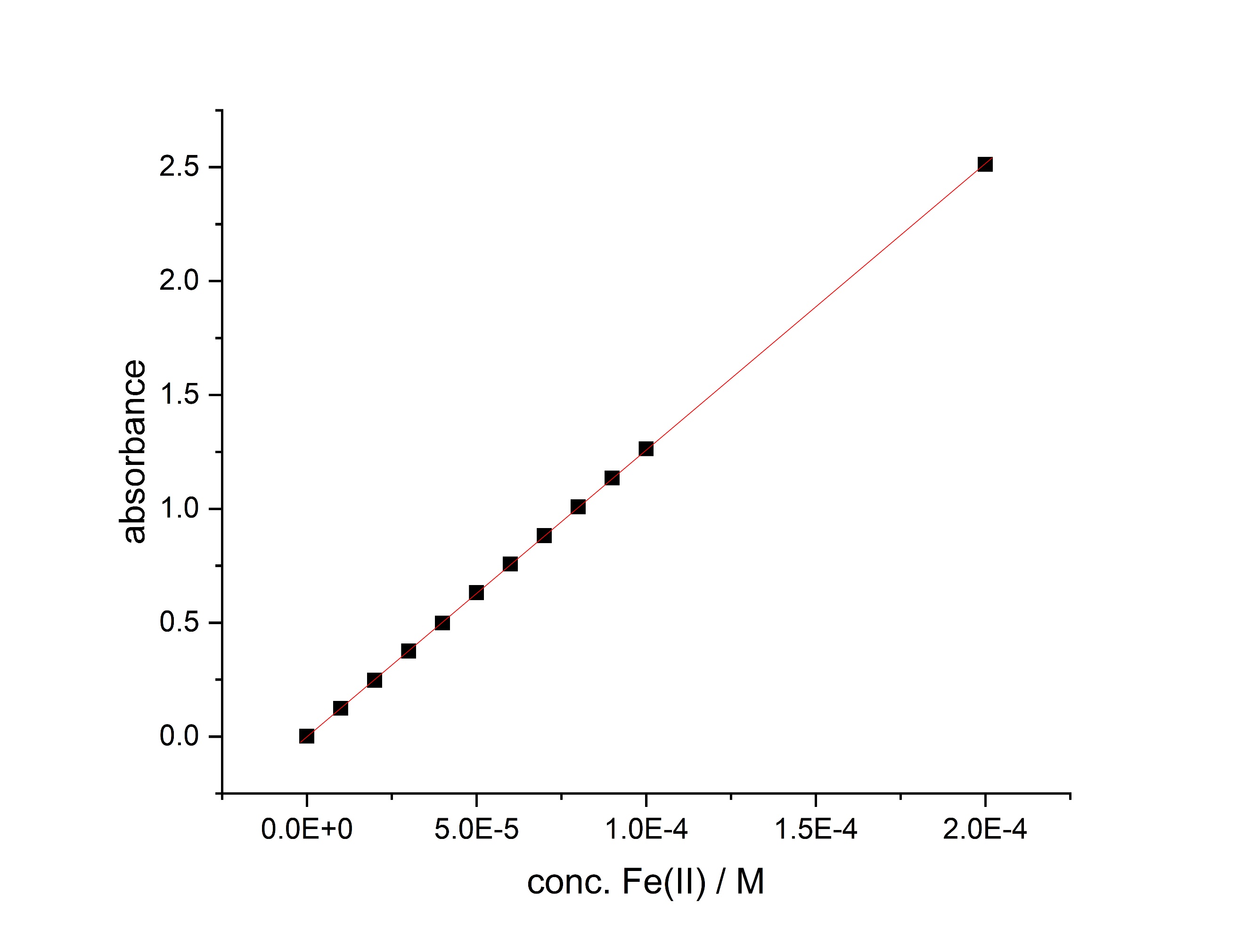


Figure S4 | Calibration of Fe(II)SO_4_ for the determination of the Fe(II)-concentration from chemical actinometry experiments.

## S6 Reference measurement of Amoxicillin degradation

Figure S5 | Normalized AMX concentration profiles (c/c_0_) for 50 µM initial AMX: a) pH 7 experiments using no filter (full MPL spectrum), borosilicate glass (295 nm), nitrate (325 nm cut-off) and nitrite (405 nm cut-off) filters without photocatalyst (photolysis controls); b) dark experiments at pH 7 with and without photocatalyst (hydrolysis and sorption control).

## S7 Blueprints


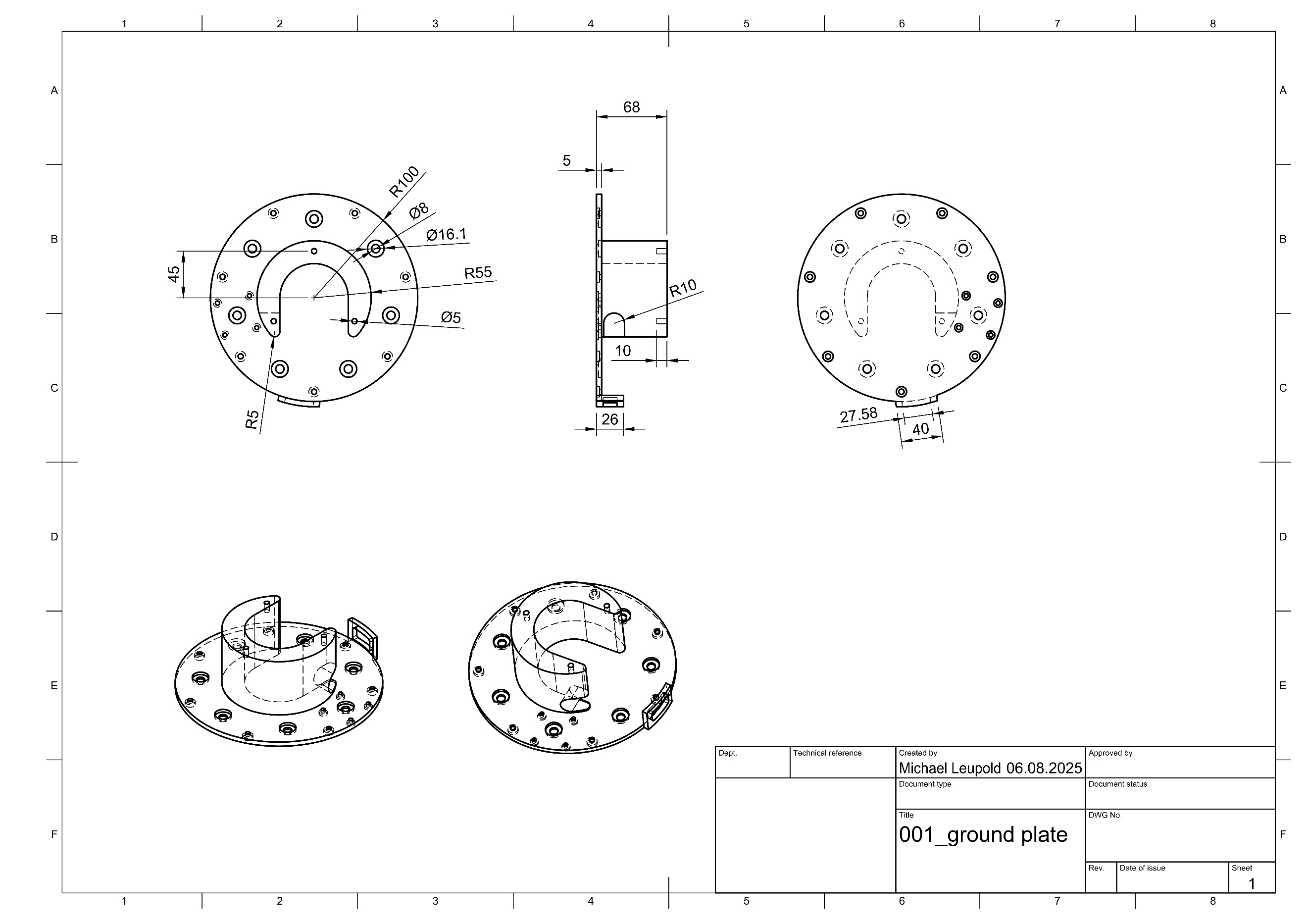


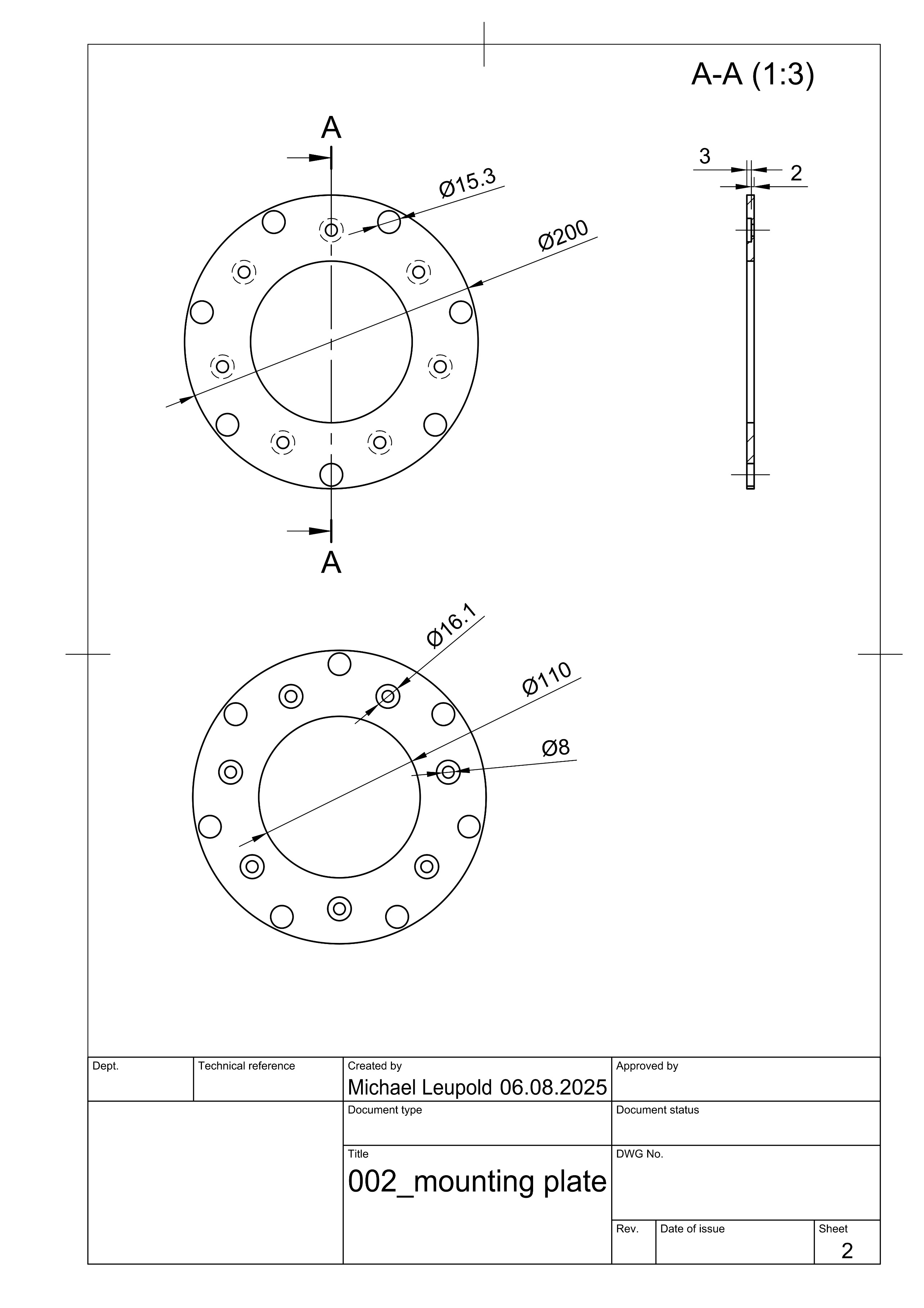

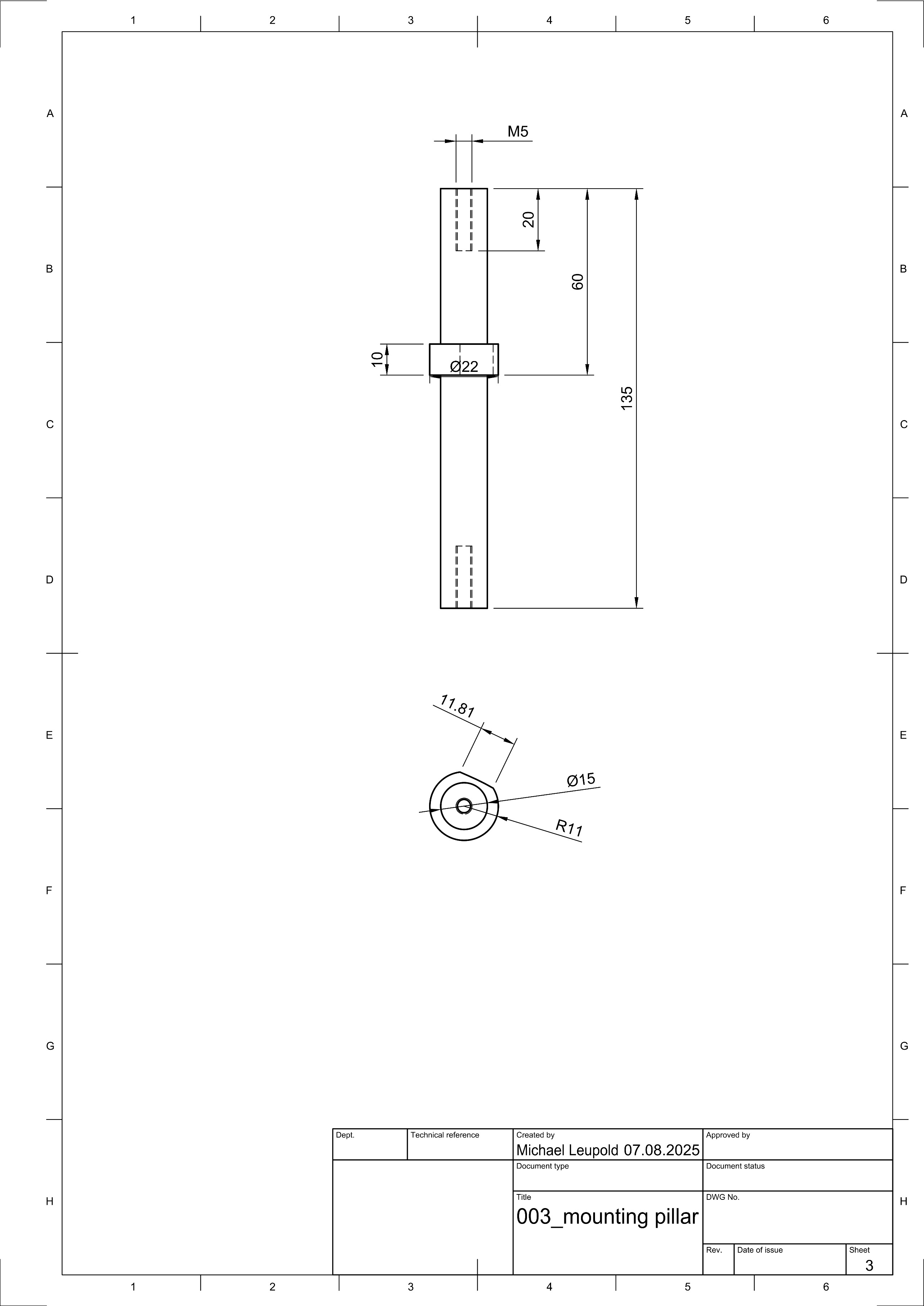

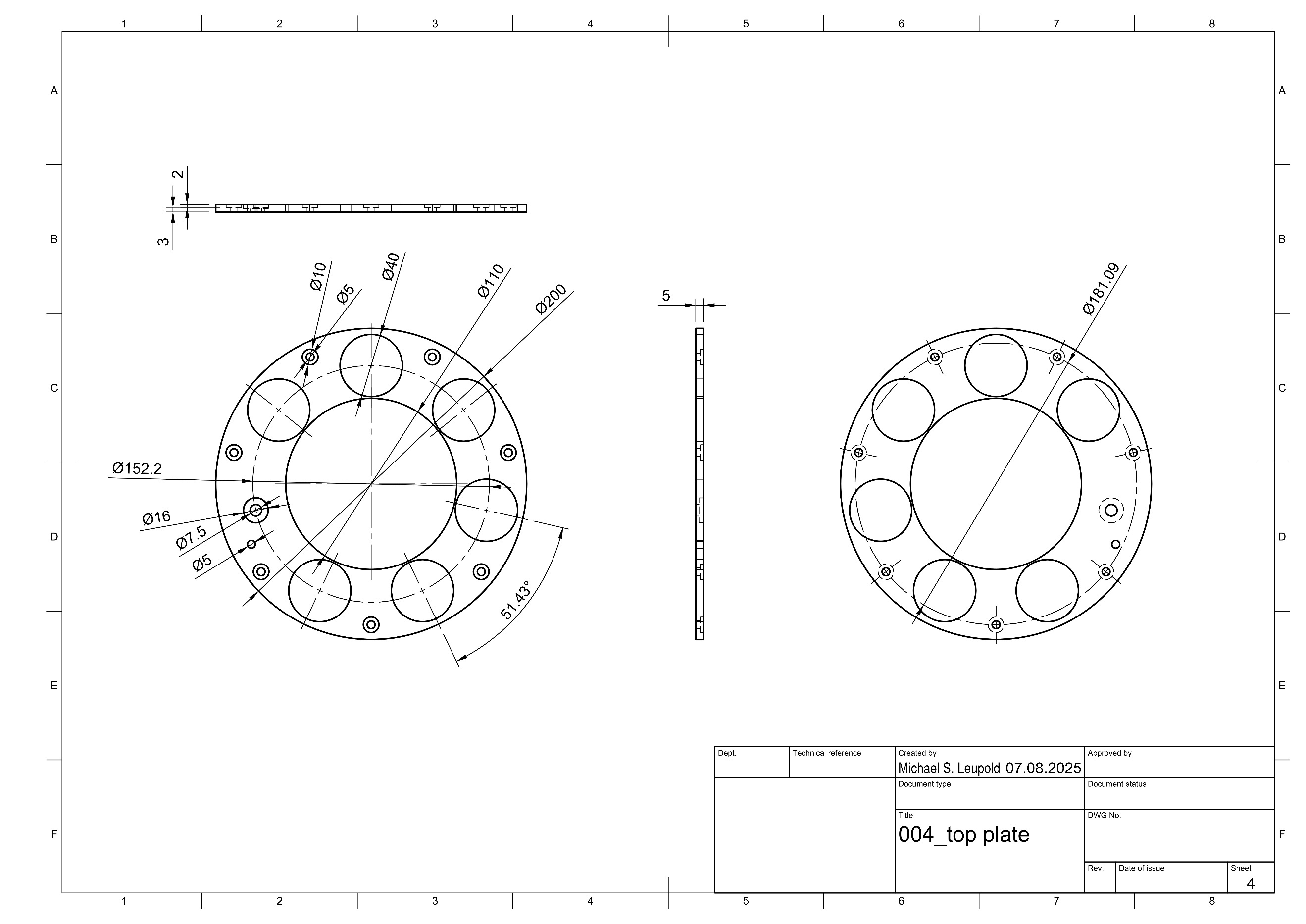

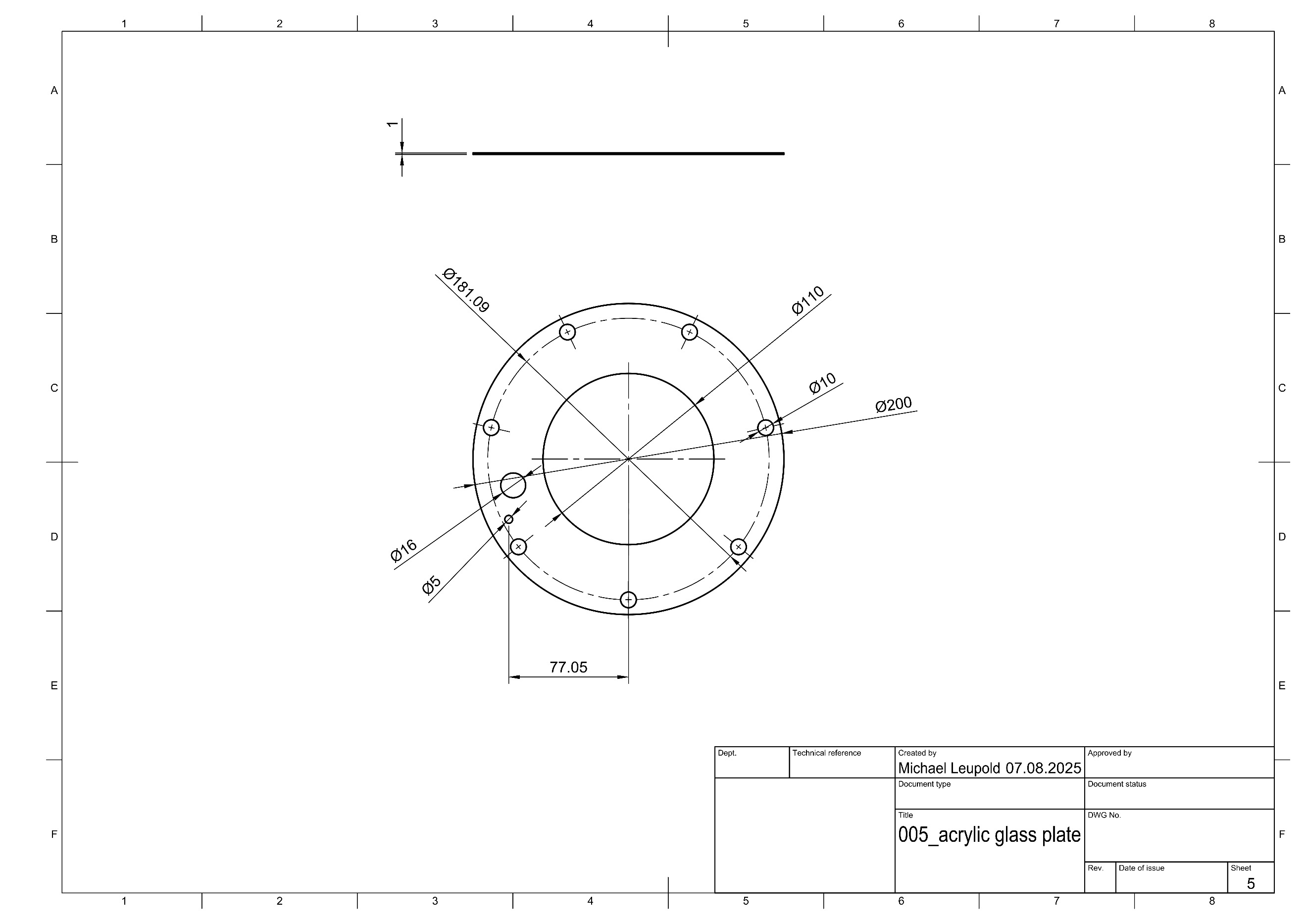

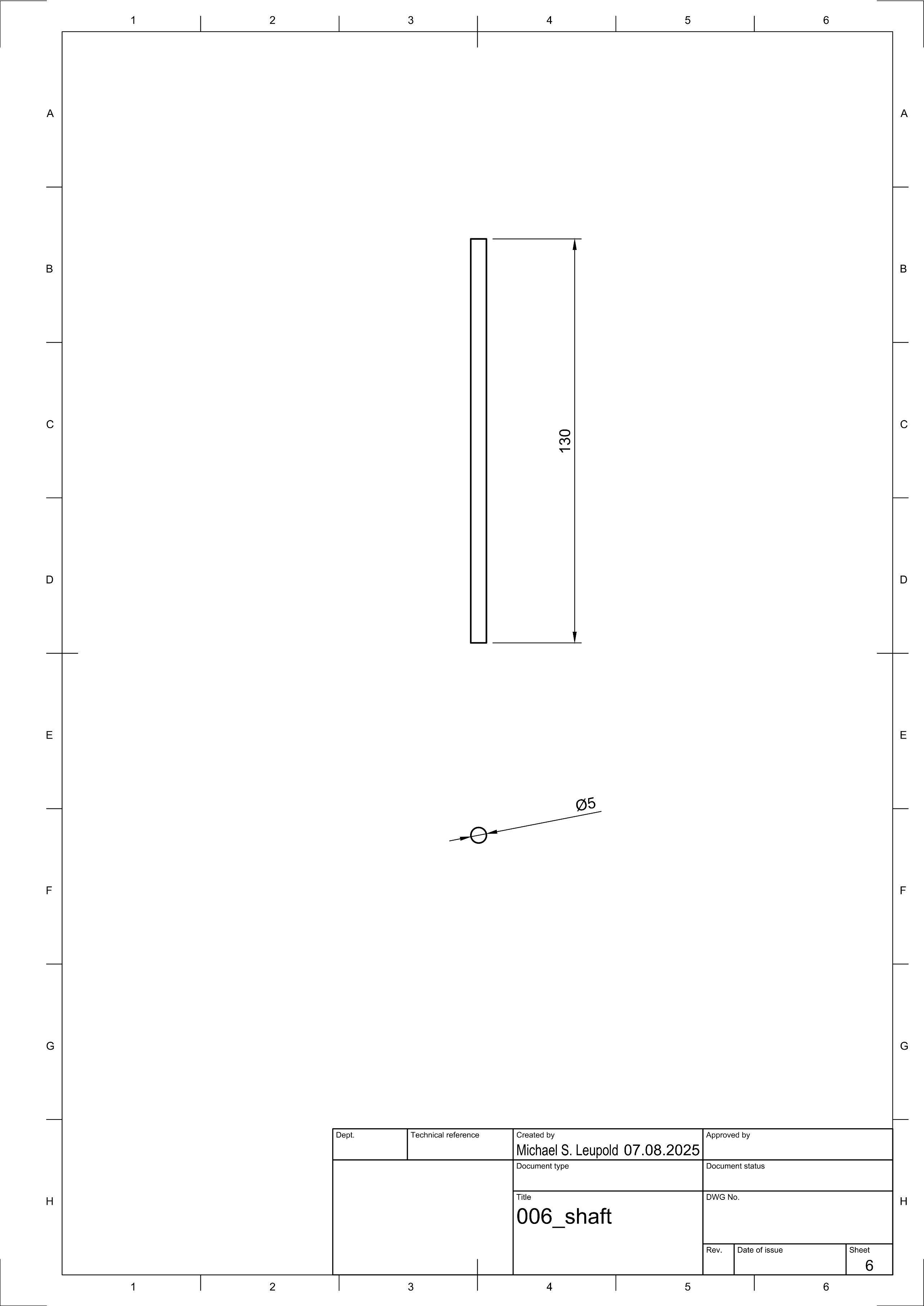

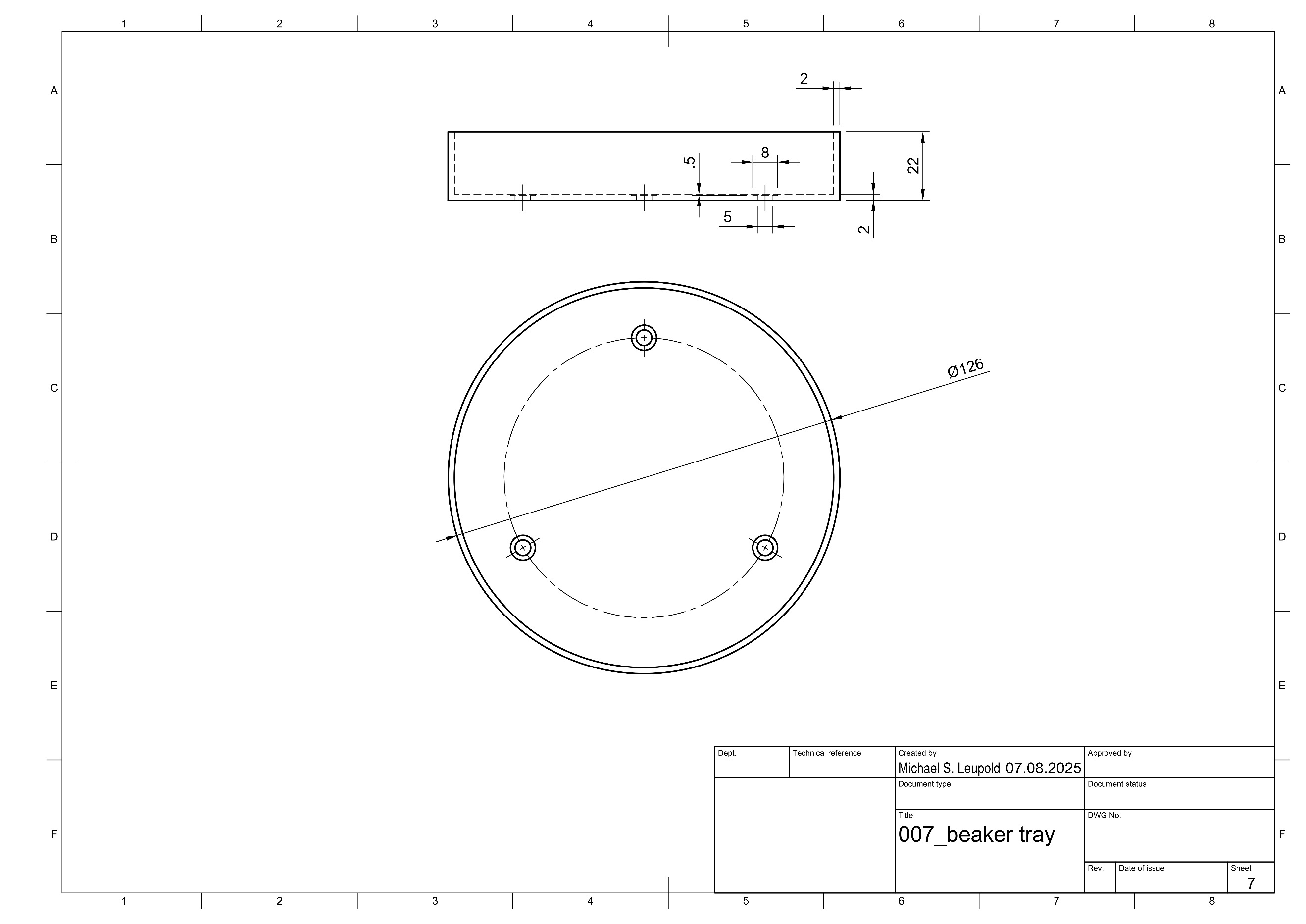

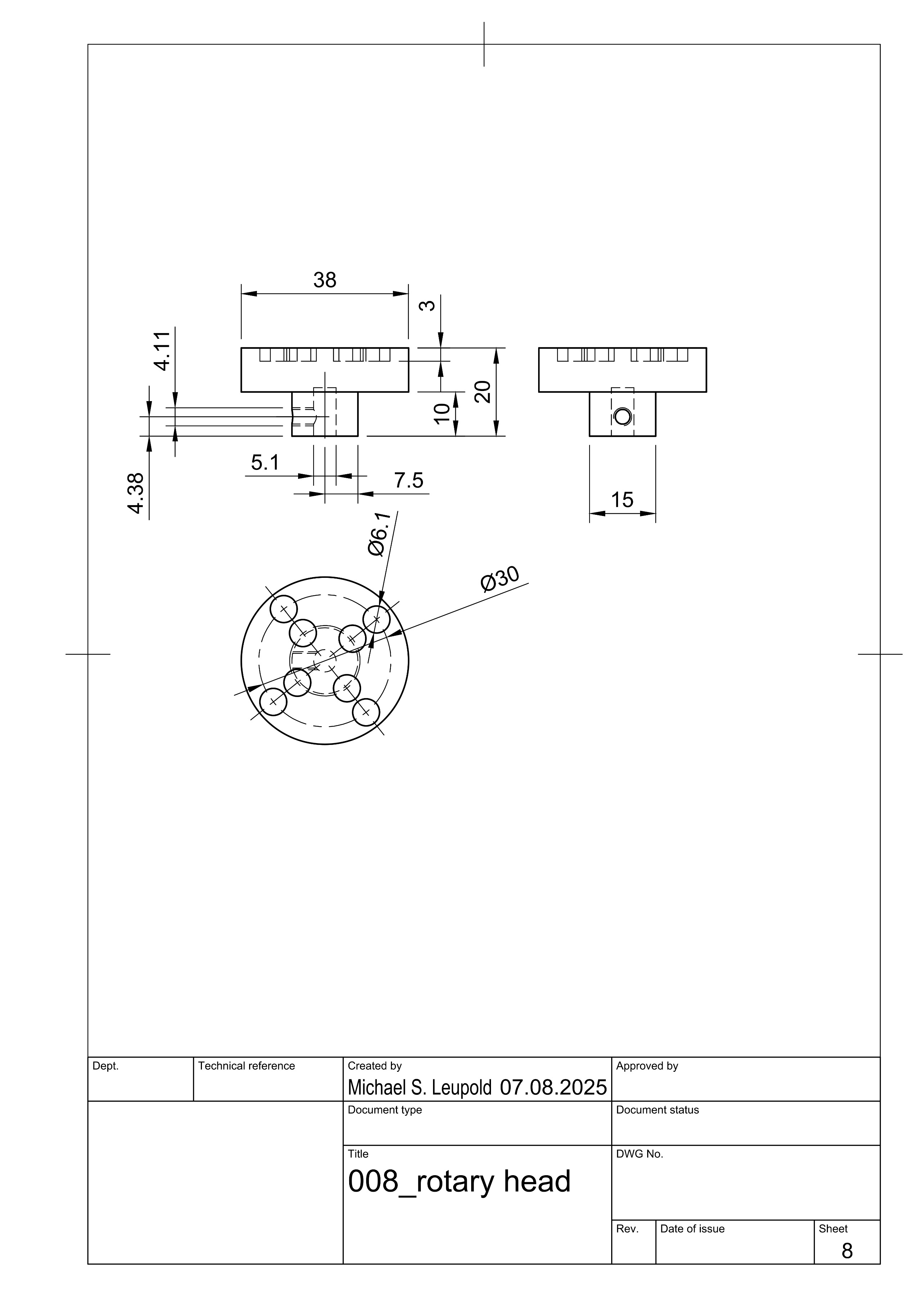

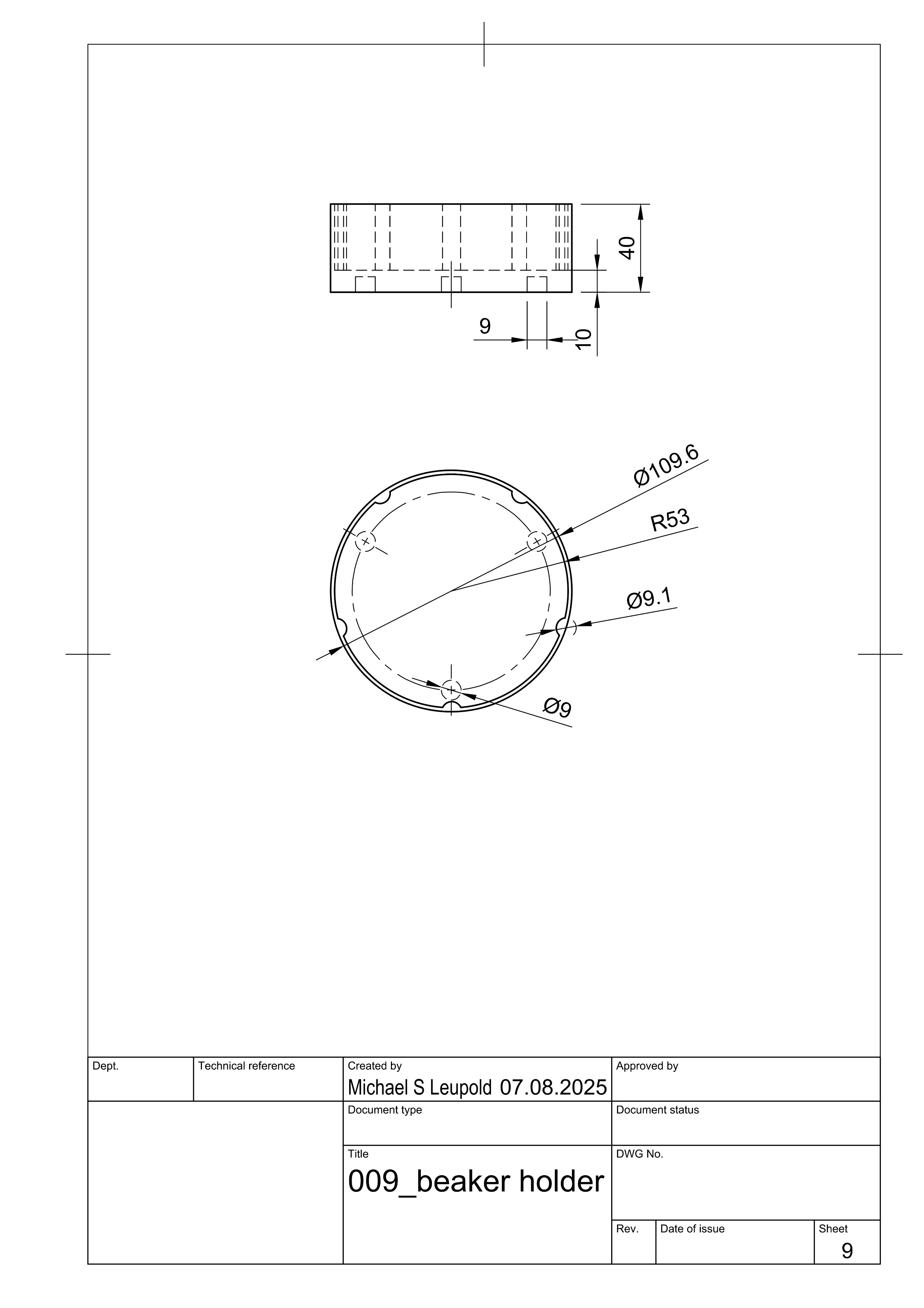

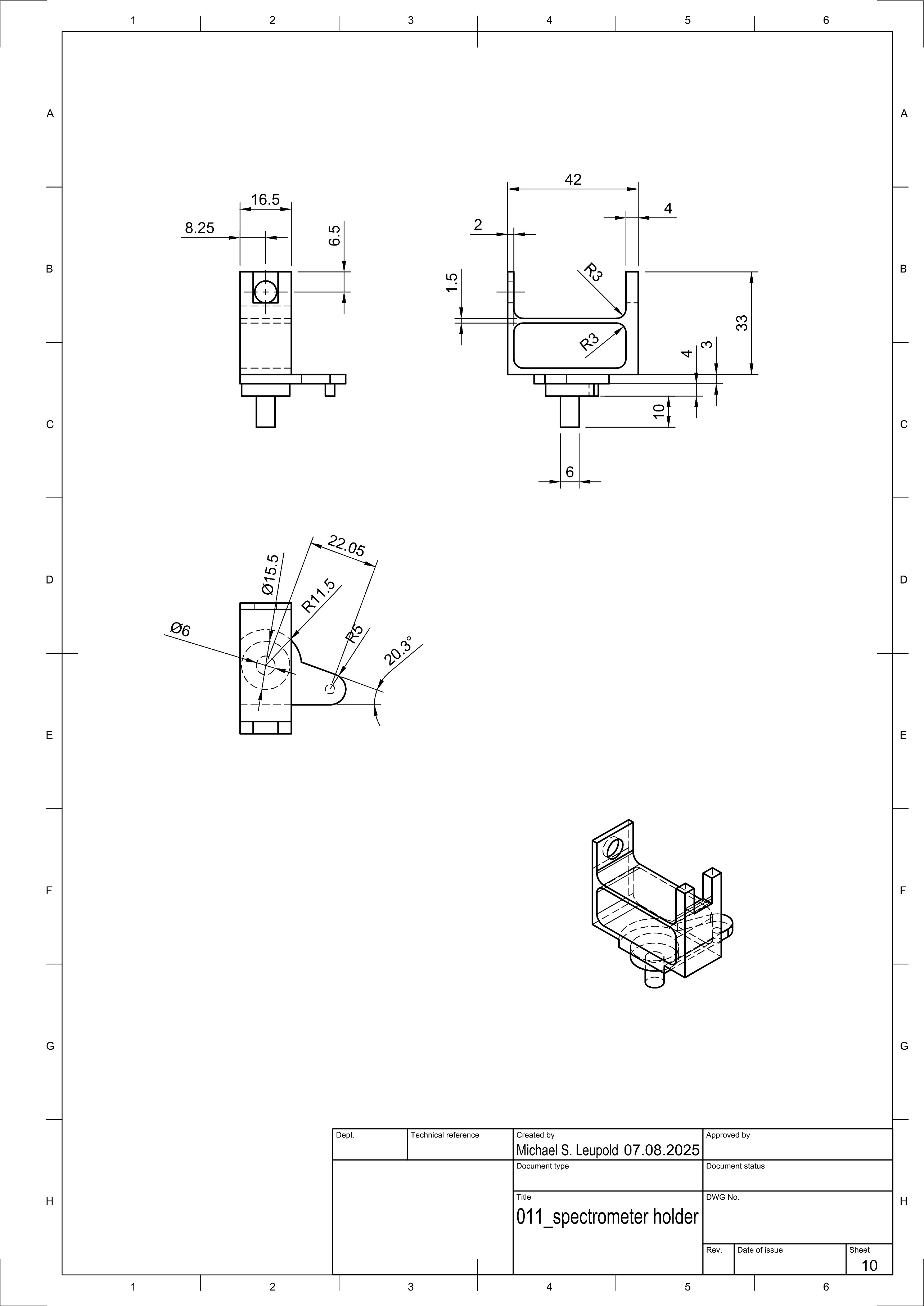

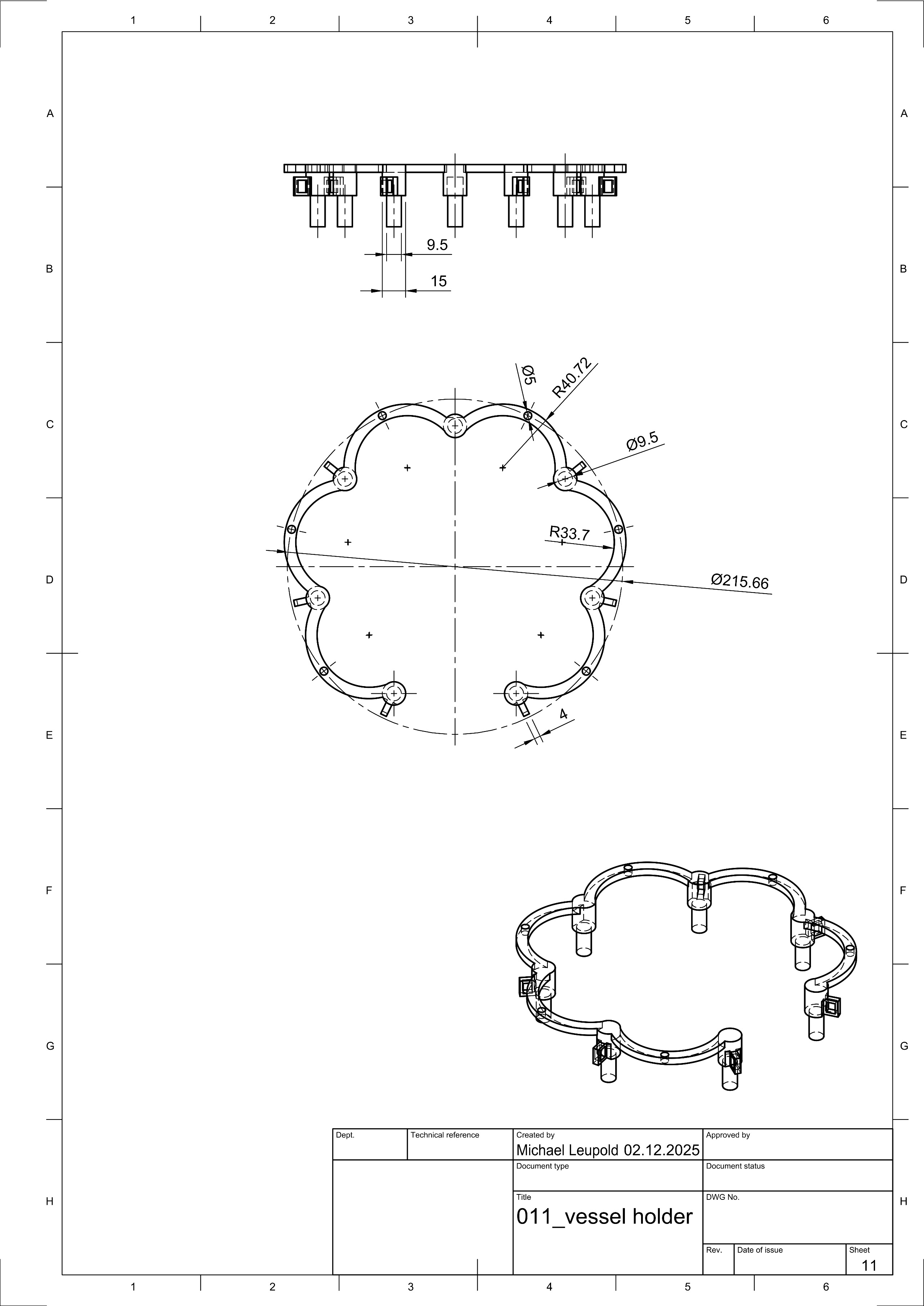

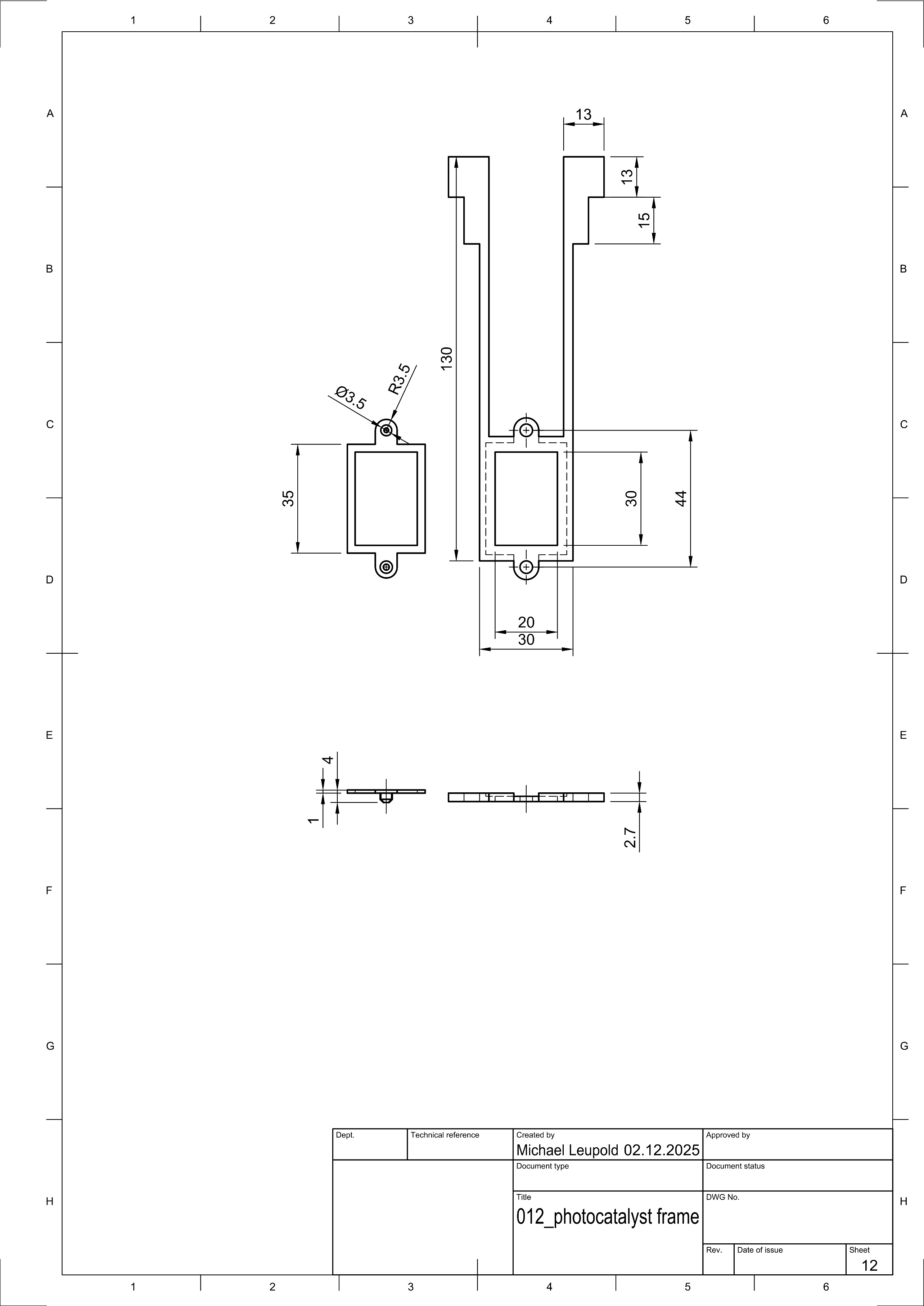

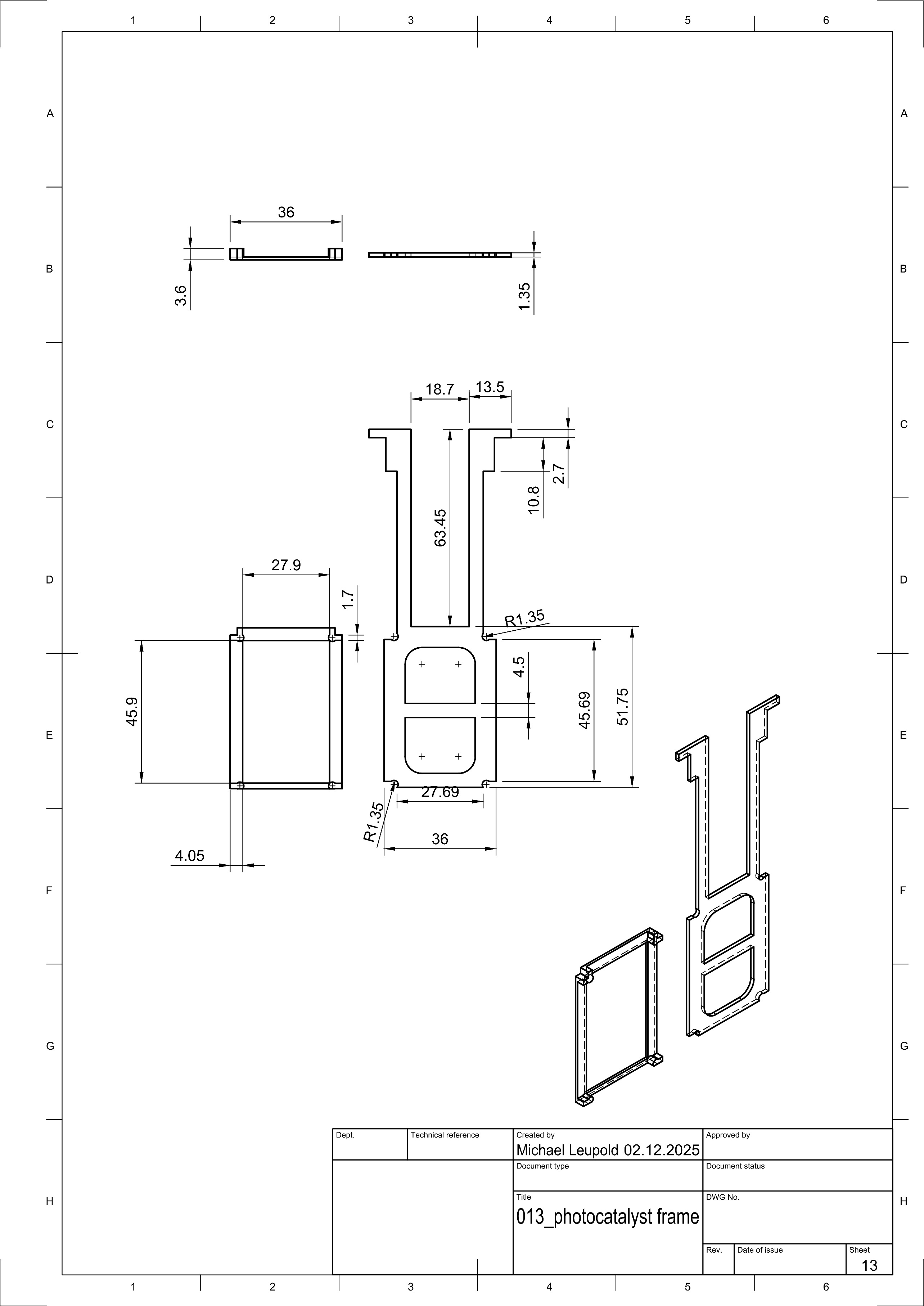


**SI References**

[1] S.M. Fonseca, S. Ahmed, T.J. Kemp, P.R. Unwin, A microelectrochemical actinometer for scanning electrochemical microscopy studies of photochemical processes, Photochemical & Photobiological Sciences 2(2) (2003) 98-103. <https://doi.org/10.1039/b209986a>.
